# A Reappraisal on the Potential Ability of Human Neutrophils to Express and Produce IL-17 Family Members *In Vitro*: Failure to Reproducibly Detect It

**DOI:** 10.3389/fimmu.2018.00795

**Published:** 2018-04-17

**Authors:** Nicola Tamassia, Fabio Arruda-Silva, Federica Calzetti, Silvia Lonardi, Sara Gasperini, Elisa Gardiman, Francisco Bianchetto-Aguilera, Luisa Benerini Gatta, Giampiero Girolomoni, Alberto Mantovani, William Vermi, Marco A. Cassatella

**Affiliations:** ^1^Department of Medicine, Section of General Pathology, University of Verona, Verona, Italy; ^2^CAPES Foundation, Ministry of Education of Brazil, Brasilia, Brazil; ^3^Department of Molecular and Translational Medicine, Section of Pathology, University of Brescia, Brescia, Italy; ^4^Department of Medicine, Section of Dermatology and Venereology, University of Verona, Verona, Italy; ^5^Humanitas Clinical and Research Center, Rozzano, Italy; ^6^Humanitas University, Pieve Emanuele, Italy; ^7^The William Harvey Research Institute, Queen Mary University of London, London, United Kingdom

**Keywords:** neutrophils, IL-17 members, IL-17A, IL-17B, IL-17F

## Abstract

Neutrophils are known to perform a series of effector functions that are crucial for the innate and adaptive responses, including the synthesis and secretion of a variety of cytokines. In light of the controversial data in the literature, the main objective of this study was to more in-depth reevaluate the capacity of human neutrophils to express and produce cytokines of the IL-17 family *in vitro*. By reverse transcription quantitative real-time PCR, protein measurement *via* commercial ELISA, immunohistochemistry (IHC) and immunofluorescence (IF), flow cytometry, immunoblotting, chromatin immunoprecipitation (ChIP), and ChIP-seq experiments, we found that highly pure (>99.7%) populations of human neutrophils do not express/produce IL-17A, IL-17F, IL-17AF, or IL-17B mRNA/protein upon incubation with a variety of agonists. Similar findings were observed by analyzing neutrophils isolated from active psoriatic patients. In contrast with published studies, IL-17A and IL-17F mRNA expression/production was not even found when neutrophils were incubated with extremely high concentrations of IL-6 plus IL-23, regardless of their combination with inactivated hyphae or conidia from *Aspergillus fumigatus*. Consistently, no deposition of histone marks for active (H3K27Ac) and poised (H3K4me1) genomic regulatory elements was detected at the IL-17A and IL-17F locus of resting and IL-6 plus IL-23-stimulated neutrophils, indicating a closed chromatin conformation. Concurrent experiments revealed that some commercial anti-IL-17A and anti-IL-17B antibodies (Abs), although staining neutrophils either spotted on cytospin slides or present in inflamed tissue samples by IHC/IF, do not recognize intracellular protein having the molecular weight corresponding to IL-17A or IL-17B, respectively, in immunoblotting experiments of whole neutrophil lysates. By contrast, the same Abs were found to more specifically recognize other intracellular proteins of neutrophils, suggesting that their ability to positively stain neutrophils in cytospin preparations and, eventually, tissue samples derives from IL-17A- or IL-17B-independent detections. In sum, our data confirm and extend, also at epigenetic level, previous findings on the inability of highly purified populations of human neutrophils to express/produce IL-17A, IL-17B, and IL-17F mRNAs/proteins *in vitro*, at least under the experimental conditions herein tested. Data also provide a number of justifications explaining, in part, why it is possible to false positively detect IL-17A^+^-neutrophils.

## Introduction

The IL-17 family of cytokines consists of six members, namely IL-17A (usually referred to as IL-17), IL-17B, IL-17C, IL-17D, IL-17E (also known as IL-25), and IL-17F (1). After the discovery of a subtype of CD4^+^ T helper, expressing IL-17A and IL-17F (currently known as Th17 cells), plenty of studies have been published correlating Th17 cells with a wide range of physiological and pathological processes. IL-17A and IL-17F are not only the most studied but also the most closely related, since they share 50% of amino acid sequence identity, adjacent gene localization (2) and binding to the same IL-17R, in this case composed by the IL-17RA and IL-17RC subunits (1). The IL-17R group comprises, in fact, five receptor subunits, IL-17RA, IL-17RB, IL-17RC, IL-17RD, and IL-17RE (3). IL-17RA was the first to be described, is ubiquitously expressed (particularly in hematopoietic cells), and functions as a common receptor subunit used by at least four ligands, namely IL-17A, IL-17C, IL-17E, and IL-17F (3). IL-17F is often coproduced with IL-17A, so that together they can also form an IL-17F/IL-17A heterodimer (4) binding to the IL-17RA/IL17RC complex as either homodimers or heterodimers (3). IL-17A and IL-17F are proinflammatory cytokines that play key regulatory roles in host defense and inflammatory diseases. They mainly mediate immune regulatory functions by promoting the generation of proinflammatory cytokines/growth factors (including G-CSF, GM-CSF, and IL-6) and chemokines (such as CXCL8, CXCL6, and CXCL1) by epithelial and other stromal cells, which ultimately lead to the attraction and activation of neutrophils and macrophages into the inflammatory site (5), as well as to granulopoeisis (6). Although crucial in protecting the host from invasion by many types of pathogens, including bacteria and fungi (7), dysregulated IL-17A and IL-17F production can lead to the development of autoimmune diseases, such as psoriasis, multiple sclerosis, and rheumatoid arthritis (RA), as well as cancer progression (5, 8). The latter observations hence make IL-17A/F as a very important target for the development of new therapies (1, 8).

As mentioned, Th17 cells are considered the main sources of IL-17A and IL-17F. However, other innate immune cells produce these cytokines, including γδ T cells, natural killer T cells, invariant natural killer cells, Paneth cells, TCRβ^+^ natural Th17 cells, lymphoid-tissue inducer-like cells, IL-17-expressing type 3 innate lymphoid cells, and mast cells (8, 9). By contrast, it is still questionable whether human polymorphonuclear neutrophils represent sources of IL-17A or IL-17F. It is currently well established that neutrophils are crucial players in innate immune responses, not only for their capacity to perform defensive functions (10) but also for their ability to produce a large variety of cytokines (11). Concerning IL-17A and/or IL-17F, in 2010, we reported that highly purified populations of human neutrophils (>99.7%), incubated for up to 20 h with IFNγ and/or LPS *in vitro*, do not produce IL-17A (12). While a few papers substantially confirm our findings (13–17), the majority of the subsequent studies report that human neutrophils may represent sources of IL-17A (18–53). Experimental evidence proving that human neutrophils express IL-17A mostly, but not only, derives by immunohistochemistry (IHC) and/or immunofluorescence (IF) studies documenting IL-17A^+^-neutrophils in tissue specimens from a variety of pathological conditions (18, 19, 21, 22, 25, 27, 28, 30–32, 34, 36–39, 41–43, 45, 47–49, 51, 53). Interestingly, many of these studies focus on psoriasis (20, 25, 30, 32, 35, 49), a disease characterized by an early accumulation of neutrophils in skin lesions in which neutrophil-derived mediators (such as reactive oxygen species, granule proteins, and cytokines) may alter the homeostatic state of keratinocytes and endothelial cells (54). At the end of 2014, however, Tamarozzi et al. (13) not only reported the absence of IL-17A mRNA expression and production by highly pure (99.9%) populations of resting or activated neutrophils but also demonstrated that some of the commercial polyclonal anti-IL-17A antibodies (Abs) stain neutrophils for their non-specific recognition of various intracellular proteins different from antigenic IL-17A. Nevertheless, reports describing either IL-17A-positive neutrophils in tissue samples from diseases or *in vitro*-stimulated neutrophils as sources of IL-17A, continue to be published (20, 23, 24, 26, 29, 33, 35, 40, 44, 46, 50, 52). Based on these premises, we decided to more accurately analyze the issue of whether human neutrophils produce IL-17A, as well as other IL-17 members *in vitro*.

## Materials and Methods

### Cell Purification and Culture

Neutrophils were isolated from buffy coats of healthy donors (HDs) and manipulated under endotoxin-free conditions (12). In selected experiments, neutrophils were also isolated from peripheral blood of patients with severe psoriasis, as defined by either >10% body surface area involved, or Psoriasis Area and Severity Index score >10, or Dermatology Life Quality Index score >10 (55). After Ficoll-Paque gradient centrifugation of buffy coats or peripheral blood, followed by dextran sedimentation of granulocytes and hypotonic lysis of erythrocytes, neutrophils were isolated to reach 99.7 ± 0.2% purity by positively removing all contaminating cells using the EasySep neutrophil enrichment kit (StemCell Technologies, Vancouver, BC, Canada) (56). Neutrophils were then suspended at 5 × 10^6^/ml in RPMI 1640 medium supplemented with 10% low (<0.5 EU/ml) endotoxin FBS (BioWhittaker-Lonza, Basel, Switzerland), incubated with or without 5 µM R848, 500 µg/ml particulate β-glucan (Invivogen, San Diego, CA, USA), 100 ng/ml ultrapure LPS (from *E. coli* 0111:B4 strain, Alexis, Enzo Life Sciences, Farmingdale, NY, USA), 1 µg/ml Pam3CSK4 (Invivogen), 50 µg/ml poly(I:C) (Invivogen), 1,000 U/ml G-CSF (Myelostim, Italfarmaco Spa, Milano, Italy), 100 U/ml IFNγ (R&D Systems, Minneapolis, MN, USA), 10 ng/ml GM-CSF (Miltenyi Biotec), 5 ng/ml TNFα (Peprotech, Rocky Hill, NJ, USA), 2–20 µg/ml IL-6 (R&D Systems), 0.2–2 µg/ml IL-23 (R&D Systems), 100–500 ng/ml IL-17A (R&D Systems), 10 µg/ml anti-IL-17A neutralizing Abs (secukinumab, Novartis, Basel, Switzerland), 100 nM fMLF, 500 µg/ml curdlan (Sigma, Saint Louis, MO, USA), 20 ng/ml phorbol mysistate acetate (PMA) (Sigma), 1 µg/ml Ionomycin (Sigma), 100 µg/ml CpG oligodeoxynucleotides (ODN) (Invivogen), and 1,000 U/ml PEGylated IFNα-2a (Pegasys, Roche, Basel, Switzerland). Inactivated conidia and hyphae from *Aspergillus fumigatus* were kindly provided by prof. Luigina Romani (University of Perugia, Italy), and used at a neutrophil-fungi ratio of 1:5 for *A. fumigatus* conidia and 1:1 for *A. fumigatus* hyphae, as previously described (57). Neutrophils were plated either in 6/24-well tissue culture plates or in polystyrene flasks (from Greiner Bio-One, Kremsmünster, Austria) for culture at 37°C, 5% CO_2_ atmosphere. After the desired incubation period, neutrophils were either processed for chromatin immunoprecipitation (ChIP) experiments or collected and spun at 300 × *g* for 5 min for other types of assays. In the latter case, cell-free supernatants were immediately frozen in liquid nitrogen and stored at −80°C, while the corresponding cell pellets were either extracted for total RNA or lysed for protein analysis. Th1 and Th17 clones (58) were kindly provided by prof. Francesco Annunziato (University of Firenze). CD4^+^ T cells were isolated by CD4^+^ T Cell Isolation Kit (Miltenyi Biotec) and stimulated for up 72 h with anti-CD3 and anti-CD28 mAbs (5 µg/ml, BD Biosciences).

### Flow Cytometry Experiments

For flow cytometry, 10^5^ neutrophils were harvested after the desired treatment, centrifuged, and suspended in 100 µl PBS containing 10% complement-inactivated human serum for FcγR blocking. Neutrophils were then stained for 15 min at T room with: APC anti-human IL-17RA/CD217 (clone 424LTS) and APC mouse IgG1к, as isotype control (clone P3.6.2.8.1) from eBioscience (San Diego, CA, USA); PE anti-human IL-17RC (clone 309822) and mouse PE IgG2B isotype control from R&D systems; PE-vio770 anti-human CD11b (clone ICRF44), FITC anti-human CD66b (clone G10F5), and PerCP-Cy5.6 anti-human CD16 (clone 3G8) from BioLegend (San Diego, CA, USA); APC anti-human CD62L (clone 145/15 Miltenyi Biotec), all at working dilutions specified in the corresponding datasheets. Sample fluorescence was then measured by MACSQuant Analyzer (Miltenyi Biotec), while data analysis performed using FlowJo software version 10 from Tree Star (Ashland, OR, USA) (59). For neutrophils of psoriatic patients, 100 µl whole blood were stained with APC anti-human IL-17RA and PE anti-human IL-17RC Abs in combination with the following mAbs: VioBlue anti-human CD14 (clone TÜK4), PE anti-human CD56 (clone AF12-7H3), PE-Vio770 anti-human CD3 (clone BW264/56), APC anti-human CD19 (clone LT19) from Miltenyi; Brilliant Violet anti-human CD45 (clone 2D1), PerCP-Cy5.5 anti-human CD16 (clone 3G8), and APC-Cy7 anti-human HLA-DR (clone L243) from BioLegend. After red cells lysis by the ammonium chloride buffer, sample fluorescence was immediately measured as previously described.

### Superoxide Anion Measurement

After isolation, neutrophils were suspended at the concentration of 2 × 10^6^ cells/ml in HBSS buffer containing 0.5 mM CaCl_2_ and 1 mg/ml glucose. Neutrophils (100 µl/well) were then distributed in a 96-well plate and incubated for 10 min at 37°C prior to the addition of 80 µM cytochrome *c*, 2 mM NaN_3_ (Sigma) and the indicated stimuli, including 20 ng/ml PMA as control. Plates were then incubated at 37°C in an automated ELx808IU microplate reader (BioTek Instruments, Inc., Winooski, VT, USA) to record cytochrome *c* reduction (*via* absorbance at 550 and 468 nm, at intervals of 5 min for 90 min). ${\text{O}}_{2}^{-}$ production was finally calculated using an extinction coefficient of 24.5 mM (60).

### Immunocitochemistry, IHC and IF

Cytospin preparations of neutrophils (61) previously cultured with the indicated stimuli were stained by ematoxylin and eosin for morphological evaluation. After coverslip removal, specimens were rehydrated through a scale of alcohols, with endogenous peroxidase activity blocked by treatment with 0.3% H_2_O_2_ in methanol for 20 min. Anti-human IL-17A (AF-317-NA), IL-17B (AF1248), and CXCL8 (AF-208) goat IgG pAbs from R&D Systems were 1:50 diluted, added to specimens for 60 min and then revealed using the goat-on-Rodent HRP-polymer (Biocare Medical, Pacheco, CA, USA) followed by diaminobenzidine. Omission of the primary antibody, as well as isotype control staining, was also performed as negative controls. For IL-17A and IL-17B tissue immunostaining, 4-µm tissue sections from two FFPE cases of pustular psoriasis were deparaffinized and rehydrated through a scale of alcohols. Endogenous peroxidase activity was then blocked by treatment with 0.3% H_2_O_2_ in methanol for 20 min. Epitope retrieval was performed using a microwave oven in 1.0 mM EDTA buffer (pH 8.0), for 3 cycles of 5 min at 750 W. IL-17A and IL-17B were diluted 1:50 and revealed using the goat HRP-polymer (IHC) or the horse anti-goat IgG biotinylated (Vector Laboratories, Peterborough, UK) followed by streptavidin-FITC (Southern Biotech, Birmingham, AL, USA). DAPI was used for counterstaining. For double IHC, anti-IL-17A and IL-17B Abs were diluted 1:500, and after revelation (as detailed above), anti-CD66b Abs (diluted 1:80 from BioLegend) were added to the sections. Mach4 AP polymer was used as secondary antibody followed by Ferangi Blue as chromogen. Ematoxylin was used for counterstaining.

### Cytokine Production

Cytokine concentrations in cell-free supernatants and cell lysates were measured by commercial enzyme-linked immunosorbent (ELISA) kits, specific for: IL-17A (DY317 from R&D systems and 88-7176 from eBioscience), IL-17A/F (88-7117, eBioscience), IL-17B [ABKA2223 from Abnova (Taipei, Taiwan) and ab171344 from Abcam (Cambridge, United Kingdom)], IL-17F (887478, eBioscience), and CXCL8 (Mabtech, Nacka Strand, Sweden). ELISA detection limits were 4 pg/ml (eBioscience) and 15.6 pg/ml (R&D) for IL-17A, 30 pg/ml for IL-17A/F, 24 pg/ml (Abnova) and 10 pg/ml (Abcam) for IL-17B, 16 pg/ml for IL-17F, and 8 pg/ml for CXCL8.

### Reverse Transcription Quantitative Real-Time PCR (RT-qPCR)

Total RNA was extracted from neutrophils by the RNeasy Mini Kit (Qiagen, Venlo, Limburg, Netherlands), as previously detailed (62). To completely remove any possible contaminating DNA, an on-column DNase digestion with the RNase-free DNase set (Qiagen) was performed during total RNA isolation. Total RNA was then reverse-transcribed into cDNA using Superscript III (Life Technologies, Carlsbad, CA, USA) and random hexamer primers (Life Technologies), while qPCR was carried out using Fast SYBR^®^ Green Master Mix (Life Technologies). Sequences of gene-specific primer pairs (Life Technologies) are listed in Table S1 in Supplementary Material. Data were calculated by Q-Gene software^1^ and expressed as mean normalized expression units after GAPDH normalization (63).

### Immunoblotting Experiments

Total neutrophil proteins were recovered from protein-rich flow-through solutions after the first centrifugation step of the RNeasy mini kit (Qiagen) procedure used for total RNA extraction, as previously described (62). Protein-rich flow-through from neutrophils were then immunoblotted by standard procedures using the anti-human IL-17A (AF-317-NA) and IL-17B (AF1248) goat IgG pAbs from R&D Systems; anti-human phospho-STAT3 (Tyr705) rabbit pAbs (#9131, Cell Signaling, Beverly, MA, USA); anti-human STAT3 rabbit pAbs (sc-482, Santa Cruz Biotechnology, Dallas, TX, USA), and anti-human β-actin mAbs (A5060 from Sigma). Blotted proteins were detected by using the Odyssey infrared imaging system (LI-COR Biosciences, Lincoln, NE, USA) (62).

### ChIP Assays

Chromatin immunoprecipitation experiments were performed exactly as previously described (62). Briefly, nuclear extracts from 2 × 10^6^ neutrophils or Th17 cell lines were immunoprecipitated using 1 µl anti-H3K4me1 (ab8895) and anti-H3K27Ac (ab4729) pAbs (both from Abcam, Cambridge, United Kingdom). Coimmunoprecipitated material was subjected to qPCR analysis using the specific promoter primers (purchased from Life Technologies) listed in Table S2 in Supplementary Material. Data from qPCR were expressed as percentage over input DNA and are displayed as mean ± SEM.

### ChIP-seq

Purified DNA from H3K27Ac and H3K4me1 ChIP assays (performed as described in the previous paragraph) was adapter-ligated and PCR-amplified for sequencing on HiSeq2000 platform (Illumina, Cambridge, UK) using TruSeq DNA Library Prep Kit (Illumina). After sequencing, reads were quality-filtered according to the Illumina pipeline. Single end (51 bp) reads were then mapped to the human genome (Genome Reference Consortium GRCh37, Feb/2009) using BOWTIE v1.0.0 (64). Only reads with no more than two mismatches (when compared to the reference genome) were converted to tag directories using HOMER’s module known as “makeTagDirectory,” and then converted to BedGraph format using HOMER’s module known as “makeUCSCfile,” to be finally normalized to 10^7^ total tag counts. ChIP-seq signals were visualized using Integrative Genomics Viewer. For H3K4me1 and H3K27Ac ChIP-seqs of Th17 cells, 36 bp reads, already filtered and mapped, were downloaded from database of the “roadmap epigenomics project”^2^ (NIH Epigenomics Roadmap Initiative). Aligned reads were then converted to BedGraph format and normalized to 10^7^ total tag counts.

### Gene Expression Data Set of Normal Hematopoietic Stem and Progenitor Cells

Gene expression profiles of cells from normal bone marrow at different stages of human granulopoiesis were downloaded from Gene Expression Omnibus Database (GEO number: GSE42519) (65). Gene expression means and SEs were calculated from the values of the biological replicates present in the GEO database.

### Statistical Analysis

Data are expressed as mean ± SEM or mean ± SD. Statistical evaluation was performed by using, depending on the experiment type, Student’s *t*-test or two-way ANOVA followed by Bonferroni’s *post hoc* test. *P* values <0.05 were considered as statistically significant.

### Study Approval

Human samples were obtained following informed written consent by both HDs and psoriatic patients. This study was carried out in accordance with the recommendations of Ethic Committee of the Azienda Ospedaliera Universitaria Integrata di Verona (Italy). All the experimental protocols were approved by the Ethic Committee and all subjects gave written informed consent in accordance with the Declaration of Helsinki.

## Results

### Human Neutrophils Incubated With a Variety of Agonists *In Vitro* Do Not Express IL-17 Members at Both mRNA and Protein Levels

**Figure 1:**
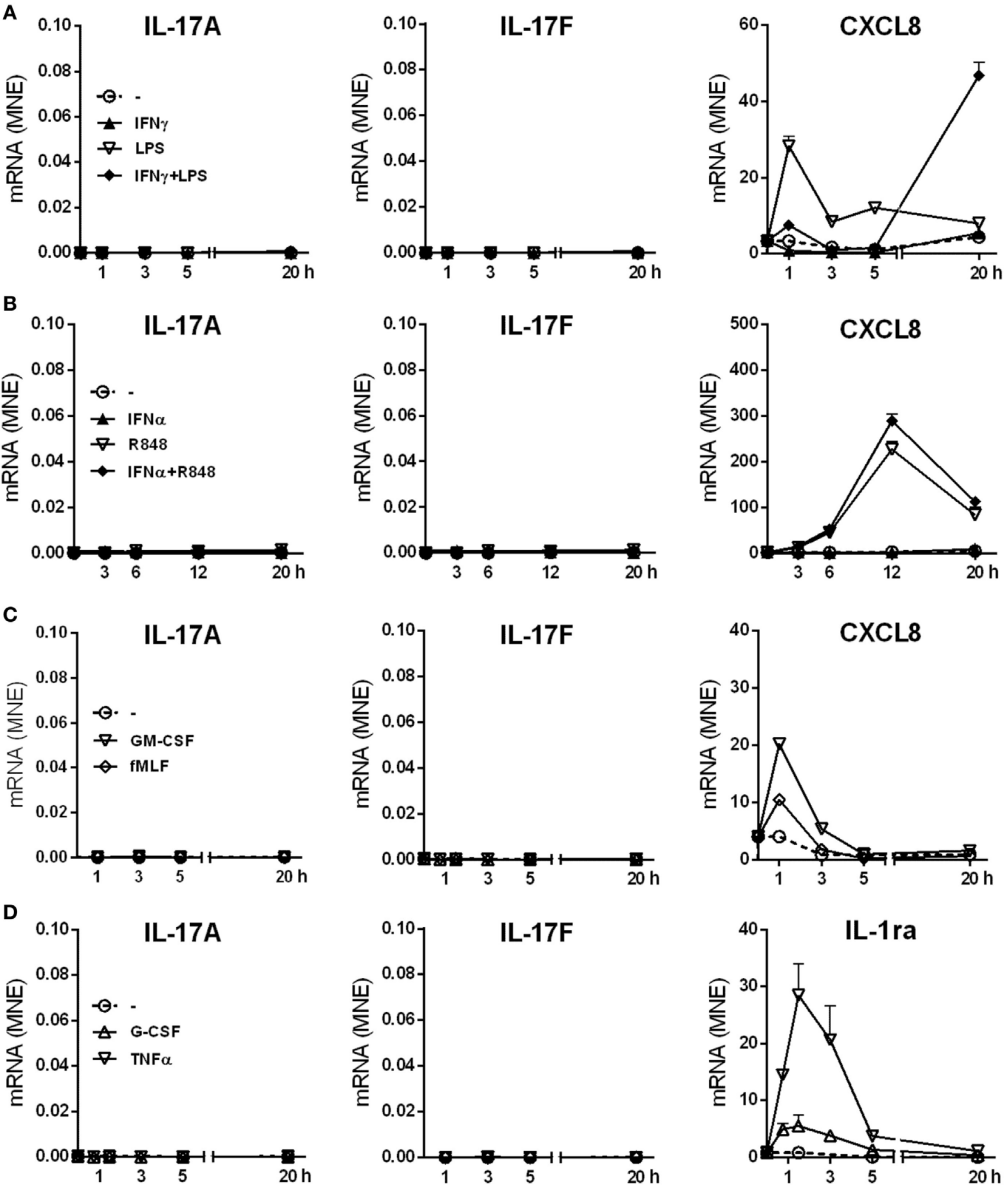
IL-17A, IL-17F, CXCL8, and IL-1ra mRNA expression levels in human neutrophils activated by a variety of stimuli. Human neutrophils were cultured at 5 × 10^6^/ml for up to 20 h with **(A)** 100 U/ml IFNγ and/or 100 ng/ml LPS; **(B)** 1,000 U/ml IFNα and/or 5 µM R848; **(C)** 10 ng/ml GM-CSF or 100 nM fMLF; **(D)** 1,000 U/ml G-CSF or 5 ng/ml TNFα. IL-17A, IL-17F, CXCL8, and IL-1ra mRNA expression was evaluated by reverse transcription quantitative real-time PCR (RT-qPCR) and data depicted as mean normalized expression (MNE) units after GAPDH mRNA normalization. The experiments depicted in each panels **(A–D)** are representative of at least three ones with similar results. Error bars stand for SEs calculated from triplicate qPCR reactions.

We have previously shown that human neutrophils (>99.7% purity), incubated with 100 U/ml IFNγ and/or 100 ng/ml ultrapure LPS for up to 20 h *in vitro*, do not produce IL-17A protein (12). Additional RT-qPCR experiments not only confirmed our previous data (Figure 1A) but also revealed that other agonists, including 5 µM R848 and/or 1,000 U/ml IFNα (Figure 1B), 10 ng/ml GM-CSF, 100 nM fMLF (Figure 1C), 1,000 U/ml G-CSF, and 5 ng/ml TNFα (Figure 1D), similarly fail to induce an accumulation of transcripts encoding IL-17A (Figures 1A–D, left panels), IL-17F (Figures 1A–D, middle panels), IL-17B, IL-17C, IL-17D, and IL-17E (data not shown) in neutrophils. LPS and/or IFNγ, R848 and/or IFNα, GM-CSF or fMLF, however, were found to modulate the expression of CXCL8 mRNA (Figures 1A–C, right panels), while G-CSF or TNFα modulated that of IL-1ra mRNA (Figure 1D, right panel), as expected (62, 66, 67). Consistent with the gene expression data, neither IL-17A, IL-17F (Table 1) nor IL-17A/F and IL-17B (data not shown) proteins could be detected in supernatants harvested from neutrophils incubated for 20 h with the stimuli used for the experiments shown in Figure 1, as well as with 500 µg/ml β-glucan, 500 µg/ml curdlan, 1 µg/ml Pam3CSK4, 50 µg/ml poly(IC), and 100 µg/ml CpG ODN. Noteworthy, we used ELISA kits from two different commercial sources (see Materials and Methods) for either IL-17A or IL-17B, in both cases giving equivalent information. On the other hand, stimulus-dependent levels of CXCL8 could be measured in supernatants from our stimulated neutrophils, indicating that agonists were effective and cells fully responsive (Table 1). In any case, validity of both IL-17 primers and ELISA kits was demonstrated by the detection of either IL-17A, IL-17D, IL-17E, and IL-17F transcripts in human Th17, but not Th1, cell lines (Figure S1 in Supplementary Material), or IL-17A and IL-17F proteins in supernatants from CD4^+^ T cells activated with anti-CD3/anti-CD28 mAbs (Table 1). We could also detect intracellular IL-17B in lysates of human cerebral cortex (data not shown), as expected (68).

**Table 1:** Lack of IL-17A and IL-17F production by activated human neutrophils.

Stimuli	IL-17A (pg/ml)	IL-17F (pg/ml)	CXCL8 (ng/ml)
**Neutrophils**			
–	nd	nd	0.07 ± 0.05
500 μg/ml β-glucan	nd	nd	0.41 ± 0.16*
500 µg/ml curdlan	nd	nd	0.49 ± 0.02***
10 ng/ml GM-CSF	nd	nd	0.30 ± 0.13*
100 nM fMLF	nd	nd	0.33 ± 0.12*
5 ng/ml TNFα	nd	nd	1.22 ± 0.90
1 µg/ml Pam3CyS	nd	nd	10.31 ± 3.85**
50 µg/ml poly(I:C)	nd	nd	0.02 ± 0.02
100 ng/ml LPS	nd	nd	0.89 ± 0.22**
5 µM R848	nd	nd	9.47 ± 3.35**
100 µg/ml CpG ODN	nd	nd	5.57 ± 1.1***
100 U/ml IFNγ	nd	nd	0.10 ± 0.04
100 U/ml IFNγ + 100 ng/ml LPS	nd	nd	2.51 ± 1.1**
**CD4^+^ T cells**			
–	nd	nd	2.51 ± 1.1
5 µg/ml anti-CD3/CD28	739.6 ± 56.6***	948.9 ± 95.4***	172.6 ± 25.1***

*Human neutrophils (5 × 10^6^/ml) were cultured for 20 h with the indicated stimuli. CD4^+^ T cells were stimulated for up to 72 h with anti-CD3 and anti-CD28 mAbs. Cell-free supernatants were then harvested and IL-17A, IL-17F, and CXCL8 content measured by specific ELISA. Values represent the mean ± SD (*n* = 3)*.

Asterisks stand for significant increases as compared to untreated cells: *P < 0.05, **P < 0.01, ***P < 0.001, by Student’s t-test.

*nd, not detected; ODN, oligodeoxynucleotides*.

**Figure 2:**
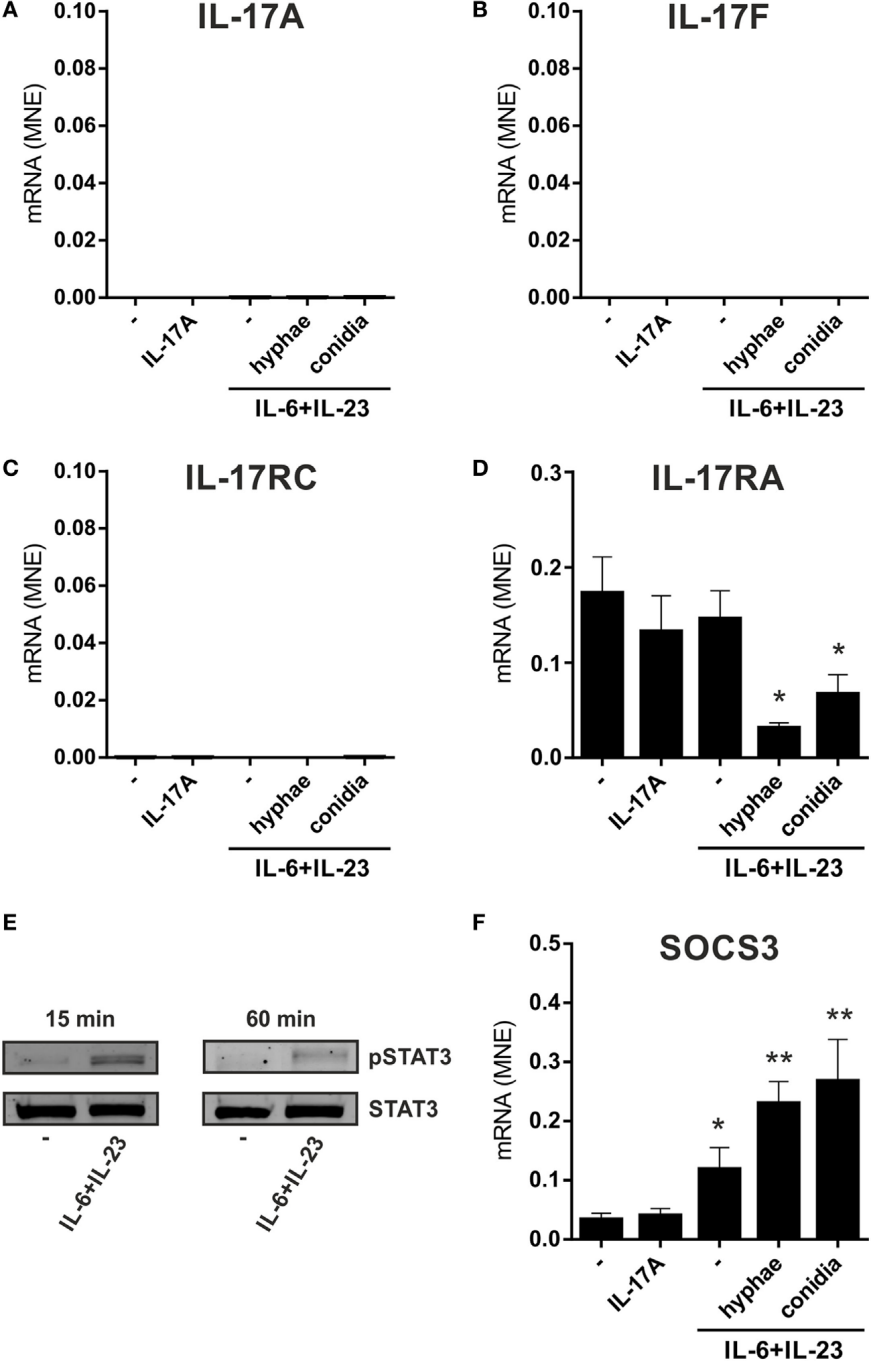
No induction of IL-17A, IL-17F, and IL-17RC mRNA expression in neutrophils incubated with IL-6 plus IL-23, in combination with inactivated *Aspergillus fumigatus* hyphae or conidia. Neutrophils (5 × 10^6^/ml) were incubated either with 100 ng/ml rIL-17A for 2 h or with or without 20 µg/ml IL-6 plus 2 µg/ml IL-23 for 1 h, prior to adding, or not, inactivated *A. fumigatus* conidia (1:5 neutrophils/conidia ratio) and hyphae (1:1 neutrophils/hyphae ratio) for additional 1 h. Neutrophils were then harvested for RNA extraction to evaluate IL-17A **(A)**, IL-17F **(B)**, IL-17RC **(C)**, IL-17RA **(D)**, and SOCS3 **(F)** mRNA expression by reverse transcription quantitative real-time PCR. Gene expression data are depicted as mean normalized expression (MNE) units after GAPDH mRNA normalization (mean ± SEM, *n* = 4). Asterisks stand for significant differences as compared to untreated cells: *P < 0.05, **P < 0.01, by Student’s *t*-test. **(E)** Immunoblot displaying STAT3 tyrosine phosphorylation in neutrophils, either untreated or cultured for 15 or 60 min with 20 µg/ml IL-6 plus 2 µg/ml IL-23 (representative experiment, *n* = 2).

**Figure 3:**
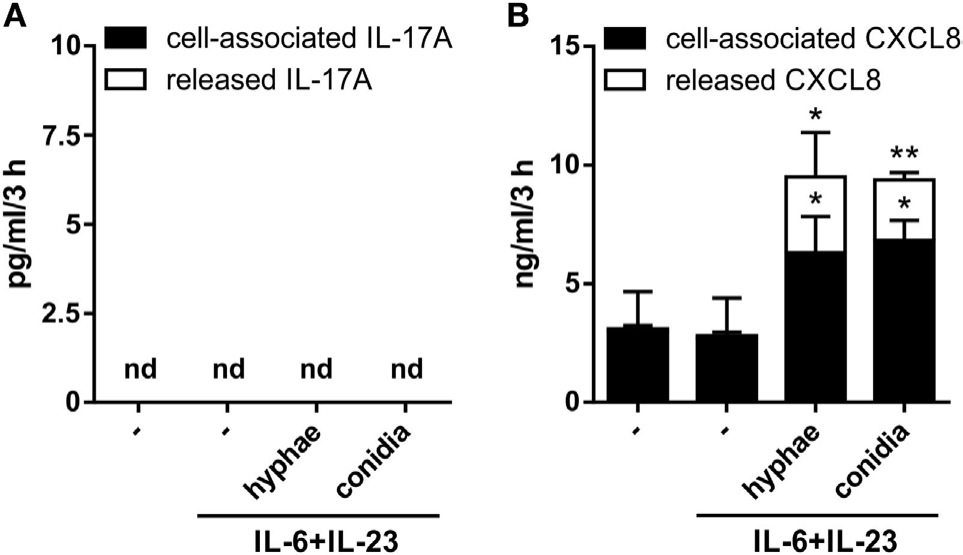
Lack of IL-17A and IL-17F production by human neutrophils activated by IL-6 plus IL-23 in combination with inactivated *Aspergillus fumigatus* hyphae or conidia. Neutrophils (5 × 10^6^/ml) were incubated with or without 20 µg/ml IL-6 plus 2 µg/ml IL-23 and then cultured for three more hours in the presence or not of inactivated *A. fumigatus* conidia and hyphae (used at 1:5 and 1:1, respectively). After incubation, IL-17A **(A)** and CXCL8 **(B)** levels were determined in cell-free supernatants and in corresponding cell pellets by specific ELISA. Values are depicted as the mean ± SD or as not detected (nd) when values were under the detection limit (*n* = 3). Asterisks stand for significant differences as compared to untreated cells: *P < 0.05, **P < 0.01, by Student’s *t*-test.

In other experiments, neutrophils were incubated for 3 h with 20 µg/ml IL-6 plus 2 µg/ml IL-23, in the presence or the absence of inactivated conidia, or hyphae, from *A. fumigatus*. These experiments were done with the purpose to mimic, as much as possible, recently described experimental conditions shown to induce not only IL-17A and IL-17F but also IL-17RC, mRNA expression (23, 24, 29, 39, 40, 44). Neutrophils were also incubated with 100–500 ng/ml IL-17A to reinvestigate (12) whether they respond to IL-17A or not. As shown in Figure 2, neutrophils treated with either IL-17A or IL-6 plus IL-23 (in the presence or the absence of inactivated *A. fumigatus* conidia/hyphae), showed neither induction of IL-17A (Figure 2A), IL-17F (Figure 2B), and IL-17RC (Figure 2C) mRNAs nor upregulation of the constitutively expressed IL-17RA transcript levels (Figure 2D). Similar results were obtained when incubation was prolonged up to 6 h (data not shown), or when neutrophils were stimulated with PMA/ionomycin after pretreatment for 1 h with IL-6 plus IL-23 (Figure S2 in Supplementary Material). Elevated levels of IL-17RC mRNAs were, however, detected in HBECs (data not shown), used as control cells (12), thus confirming that our primers were correctly designed. Importantly, the capacity of IL-6 plus IL-23 to stimulate neutrophils was evidenced by their ability to time-dependently promote STAT3 phosphorylation (Figure 2E), as well as to upregulate SOCS3 mRNA expression (Figure 2F), such an effect being potentiated by inactivated *A. fumigatus* conidia/hyphae (Figure 2F). By contrast, IL-17A-treatment influenced neither SOCS3 (Figure 2F) nor CXCL8 (data not shown) mRNA levels in neutrophils. Furthermore, no IL-17A (Figure 3A), IL-17F, or IL-17AF (data not shown) proteins were detected by ELISA either intracellularly or in supernatants harvested from neutrophils incubated with IL-6 plus IL-23, in the presence or the absence of inactivated *A. fumigatus* conidia/hyphae. Under the same experimental conditions, CXCL8 protein was newly synthesized and released by neutrophils incubated with IL-6 plus IL-23 in the presence of inactivated *A. fumigatus* conidia/hyphae, but not in their absence (Figure 3B). Finally, no IL-17A was detected in IL-6 plus IL-23-stimulated neutrophils by intracellular staining experiments (data not shown), using the anti-human IL-17A eBio64DEC17 mouse IgG1 (from eBioscience) previously shown to function under identical experimental conditions by Taylor et al. (39). We have no clues explaining why we did not reproduce the positive effects on IL-17 expression by IL-6 plus IL-23 (23, 24, 29, 39, 40), with or without inactivated *A. fumigatus* conidia/hyphae. One possibility is that the hyphal extracts from *A. fumigatus* used by Taylor and colleagues (39), but not our inactivated conidia/hyphae, contain some undefined PAMP(s) that effectively promote(s) IL-17A production/IL-17RC expression by human neutrophils.

Taken together, our data extend previous findings on the inability of human neutrophils to express IL-17 members at the mRNA and protein levels under various activating conditions (13–16). Data also confirm and extend our previous findings (12) on the inability of IL-17A to directly modify IL-17A, IL-17F, IL-17RA, IL-17RC, SOCS3, and CXCL8 gene expression in human neutrophils.

### Human Neutrophils Incubated With IL-6 Plus IL-23, in the Presence or the Absence of Inactivated *A. fumigatus* Hyphae/Conidia, Do Not Express IL-17RC

**Figure 4:**
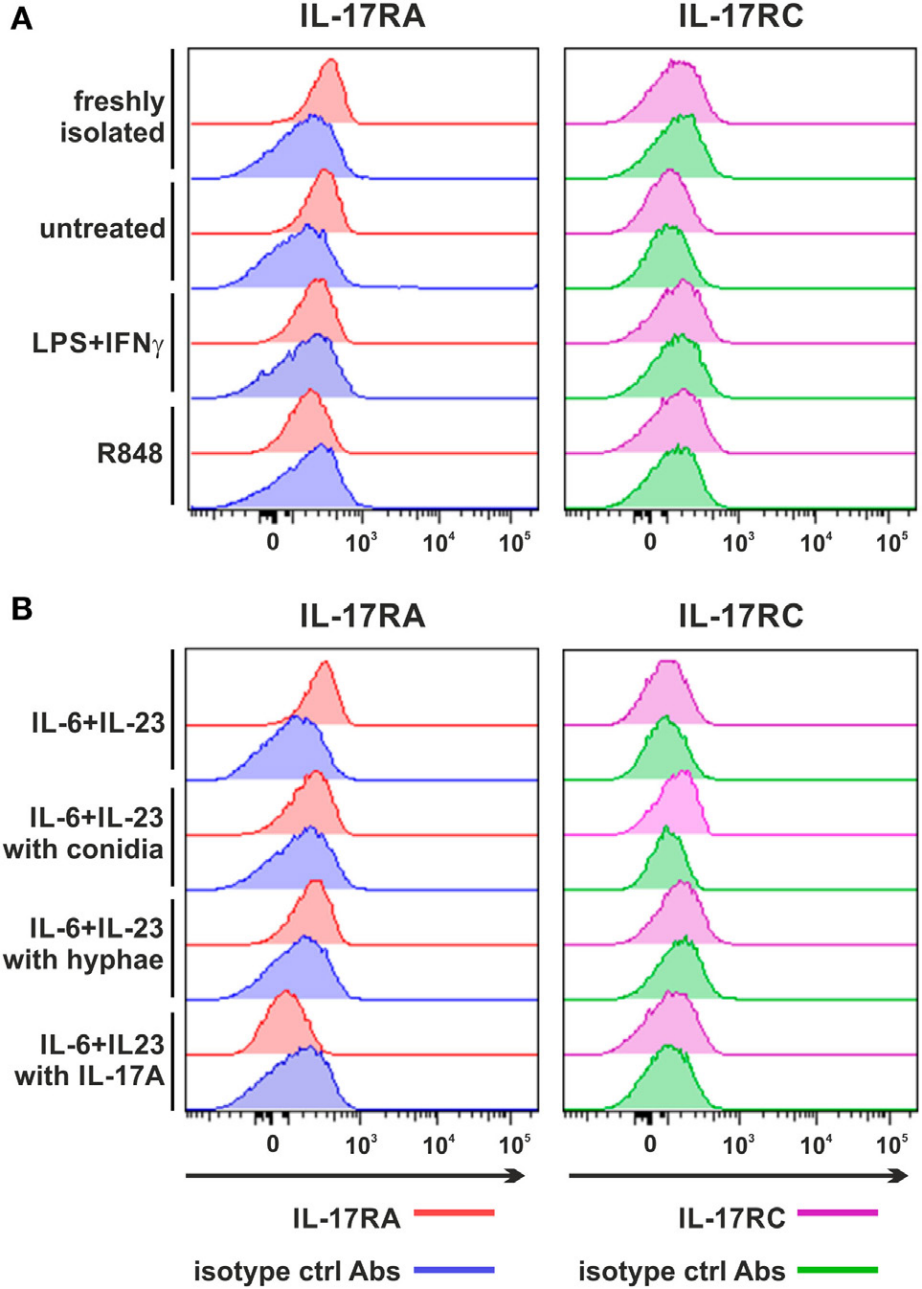
Expression of surface IL-17RA and IL-17RC in neutrophils activated under various experimental conditions. Expression of surface IL-17RA (left panel) and IL-17RC (right panel) was evaluated by flow cytometry in neutrophils either freshly isolated or cultured for 3 h without or with 100 U/ml IFNγ plus 100 ng/ml LPS, 5 µM R848 **(A)**, 20 µg/ml IL-6 plus 2 µg/ml IL-23 alone or in the presence of inactivated *Aspergillus fumigatus* conidia, hyphae or 500 ng/ml rIL-17A **(B)**. Graphs depict a representative experiment out of three independent ones with similar results. Histograms show staining by specific and isotype control Abs, respectively, for each stimulatory condition.

Flow cyometry experiments confirmed (12) that neutrophils, either freshly isolated, or incubated for 3 h in the absence, or the presence of IFNγ plus LPS (Figure 4A), display only surface IL-17RA, but not IL-17RC. No IL-17RC surface levels were also observed in neutrophils incubated with either R848 (Figure 4A), or IL-6 plus IL-23, in the latter case in the absence, or in the presence of either inactivated *A. fumigatus* conidia/hyphae, or IL-17A (Figure 4B). IL-17RA surface levels were downregulated in neutrophils treated with IFNγ plus LPS, R848 (Figure 4A) and IL-6 plus IL-23 with IL-17A (Figure 4B). In these experiments, HBEC were, again, used as positive control for both IL-17RA and IL-17RC surface expression (data not shown) (12). It should be pointed out that, for the investigation of surface IL-17RC, we have been using the same anti-IL-17RC, directly PE-conjugated, Abs used in Taylor et al.’s study (39), other than the anti-IL-17RC biotin-conjugated Abs that necessitate PE-conjugated streptavidin for detection (12), without noticing any difference between them. By the way, IFNγ plus LPS and R848 (Figure S3A in Supplementary Material), as well as IL-6 plus IL-23 in the presence of inactivated *A. fumigatus* conidia/hyphae (Figure S3B in Supplementary Material), variably modulated both CD11b and CD62L expression. All in all, data illustrate that IL-6 plus IL-23, regardless of their combination with inactivated *A. fumigatus* conidia/hyphae, and despite their capacity to upregulate SOCS3 mRNA expression (Figure 2F), do not induce the expression of IL-17RC in our hands, contradicting some studies (39, 44).

### ${\text{O}}_{2}^{-}$ Production by Neutrophils Stimulated With Inactivated *A. fumigatus* Hyphae After Preincubation With IL-6 plus IL-23 Is Not Modified by Either Exogenous IL-17A or IL-17A Inhibitors

We then measured the capacity to release ${\text{O}}_{2}^{-}$ by neutrophils preincubated with or without IL-6 plus IL-23 for 1 h, and then treated for one additional hour with inactivated *A. fumigatus* hyphae, in the presence or the absence of either IL-17A or anti-IL-17A neutralizing Abs (Figure S4 in Supplementary Material). As control, neutrophils were also stimulated with either inactivated *A. fumigatus* hyphae alone or 20 ng/ml PMA. As shown in Figure S4 in Supplementary Material, inactivated *A. fumigatus* hyphae were found to trigger a remarkable ${\text{O}}_{2}^{-}$ production by neutrophils, even though lower than PMA. However, *A. fumigatus* hyphae-stimulated ${\text{O}}_{2}^{-}$ release was not potentiated by the preincubation of neutrophils with IL-6 plus IL-23 (which, by themselves, did not trigger any ${\text{O}}_{2}^{-}$ production) (Figure S4 in Supplementary Material). Under the latter experimental conditions, addition of either IL-17A or anti-IL-17A neutralizing Abs (αIL-17A Abs) did not influence the effect of inactivated *A. fumigatus* hyphae on neutrophil-derived ${\text{O}}_{2}^{-}$ (Figure S4 in Supplementary Material), supporting the lack of induction of surface IL-17RC expression and endogenous IL-17A, respectively.

### Chromatin Organization at the IL-17A and IL-17F Genomic Loci of Human Neutrophils

**Figure 5:**
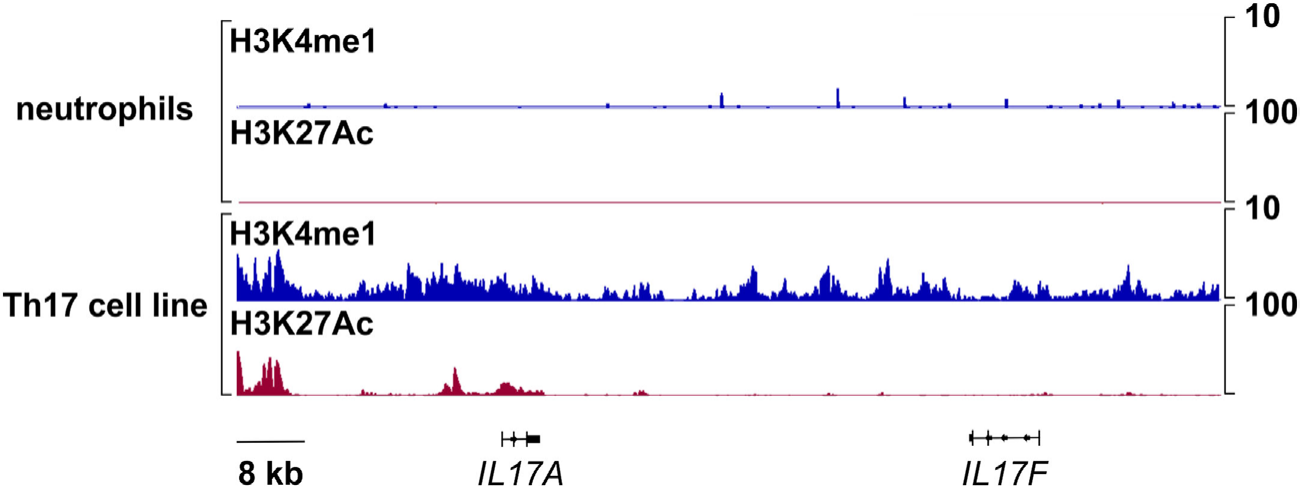
Chromatin immunoprecipitation (ChIP)-Seq profiles of H3K4me1 and H3K27Ac at the *IL17A* and *IL17F* loci in human neutrophils and Th17 cell lines. Representative snapshots depicting H3K4me1 and H3K27Ac ChIP-seqs at the *IL17A* and *IL17F* genomic loci in freshly isolated human neutrophils or, as retrieved from NIH Epigenomics Roadmap Initiative (72), in phorbol mysistate acetate/ionomycin-stimulated Th17 cell lines.

**Figure 6:**
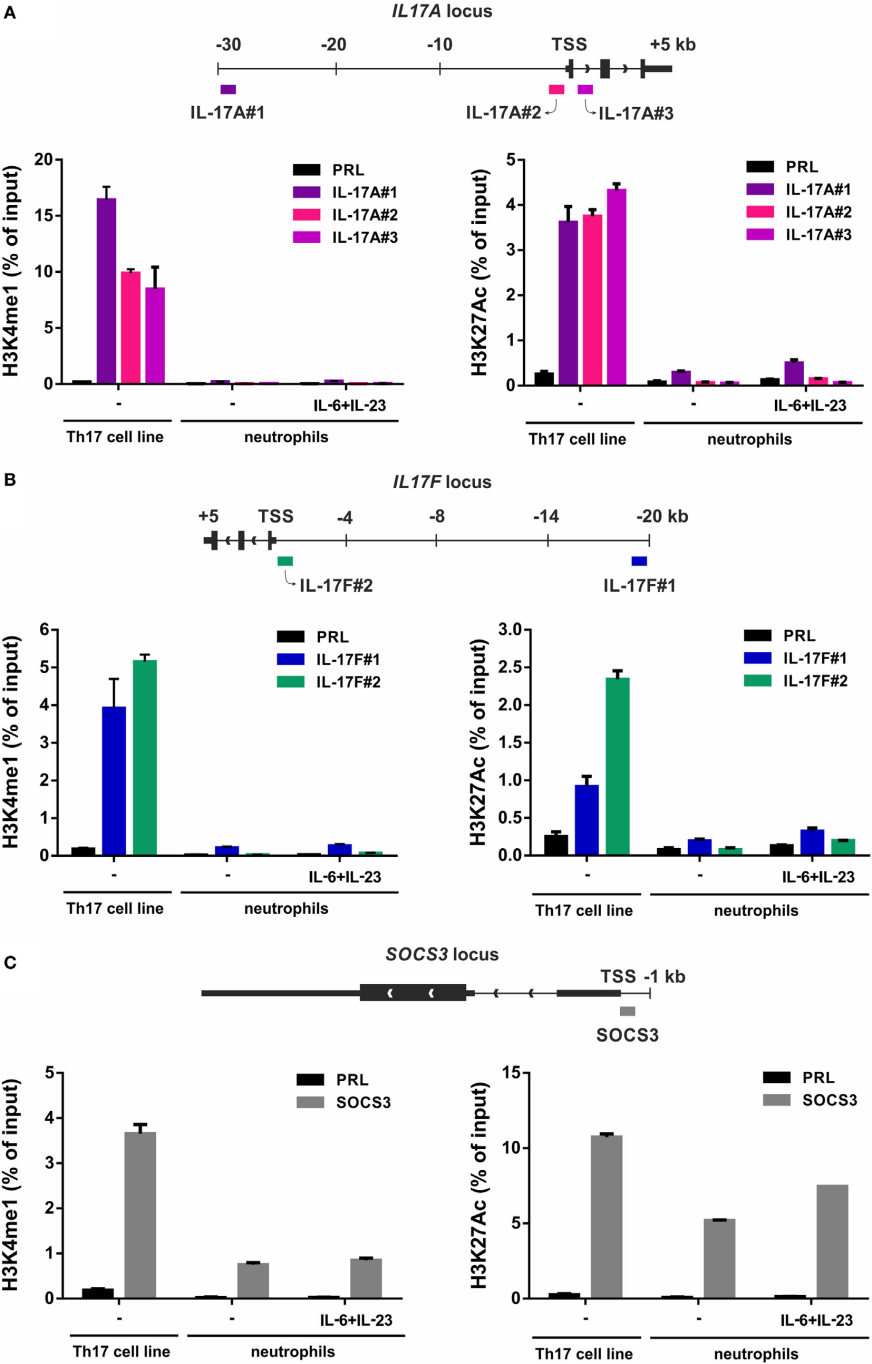
H3K4me1 or H3K27Ac levels at the IL-17A, IL-17F, and SOCS3 genomic loci of Th17 cell lines and resting/IL-6 plus IL-23-activated neutrophils. Enrichment levels of H3K4me1 (left panels) and H3K27Ac (right panels) at the IL-17A **(A)**, IL-17F **(B)**, and SOCS3 **(C)** genomic loci by chromatin immunoprecipitation (ChIP) analysis in human Th17 cell lines and neutrophils incubated for 1 h with or without 20 µg/ml IL-6 plus 2 µg/ml IL-23. **(A–C)** Schemes illustrating the positions of the designed primer pairs amplifying promoter and potential enhancer regions of IL-17A, IL-17F, and SOCS3 for ChIP analysis are depicted at the top of each panel. Coimmunoprecipitated DNA samples were expressed as percent of the total input. Panels in **(A–C)** depict a representative experiment out of two independent ones with similar results. Error bars represent SEs calculated from triplicate qPCR reactions.

Signatures of histone posttranslational modifications at a specific gene locus provide indicative elements to predict whether such a gene can be transcribed or not (69, 70). Therefore, we evaluated the presence of histone marks associated to active (e.g., H3K27Ac) and poised (e.g., H3K4me1) genomic regulatory elements (71) at the *IL17A* and *IL17F* loci of human neutrophils. Genome-wide ChIP-seq assays demonstrated that, in freshly isolated neutrophils, the entire genomic region containing *IL17A* and *IL17F* loci is completely devoid of H3K27Ac and H3K4me1 (Figure 5). By contrast, based on data available from the NIH Epigenomics Roadmap Initiative (72), multiple H3K4me1 peaks are present in the same genomic regions of PMA/ionomcyin-stimulated Th17 cells, while H3K27Ac peaks localize at the *IL17A* locus only (Figure 5). To validate the previous data, we performed H3K27Ac and H3K4me1 qPCR ChIPs using neutrophils incubated for 1 h either with or without 20 µg/ml IL-6 plus 2 µg/ml IL-23, as well as Th17 cell lines (in which IL-17A and IL-17F mRNA is constitutively transcribed), used as positive controls (Figure 6). Based on the H3K4me1 peaks from the ChIP-seqs of Th17 cell lines (72) (Figure 5), we designed specific primers amplifying potential regulatory regions at the *IL17A* and *IL17F* genomic loci, namely IL-17A#1 and IL-17F#1 for enhancers, and IL-17A#2, IL-17A#3, and IL-17F#2 for promoters (Figures 6A,B). As expected, Th17 cell lines displayed constitutively bound H3K4me1 at their IL-17A and IL-17F promoters and enhancers (Figures 6A,B, left panels). We also detected high levels of H3K27Ac at the IL-17A and IL-17F promoters and enhancers of Th17 cell lines (Figures 6A,B, right panels), in line with their constitutive expression of both IL-17A and IL-17F mRNA (data not shown). By contrast, we did not observe any H3K4me1 or H3K27Ac at the *IL17A* and *IL17F* loci of neutrophils, either under resting conditions (thus confirming the ChIP-seq data shown in Figure 5) or after incubation with IL-6 plus IL-23 (Figures 6A,B). In fact, the H3K4me1 and H3K27Ac levels at the IL-17A and IL-17F enhancers in neutrophils were similar to those ones present at the PRL promoter, a genomic region with a closed chromatin conformation in myeloid cells, herein used to determine the signal background (Figures 6A,B). Notably, measurable amounts of H3K4me1 and H3K27Ac were found at the SOCS3 promoter of neutrophils under resting conditions, as well as in Th17 cell lines (Figure 6C). Interestingly, H3K27Ac levels tended to increase in neutrophils incubated with IL-6 plus IL-23 (Figure 6C), in accordance with a supposed STAT3-dependent induction of SOCS3 mRNA (73). Taken together, data indicate that the organization of the *IL17A* and *IL17F* loci in human neutrophils is characterized by the absence of poised chromatin marks, unlike that of IL-17A- and IL-17F-producing Th17 cell lines. Data also indicate that human neutrophils do not reorganize the chromatin of the *IL17A* and *IL17F* loci in response to IL-6 plus IL-23, consistent with their inability to *de novo* accumulate IL-17A and IL-17F mRNA.

### Human Neutrophils From Patients With Psoriasis Do Not Express IL-17A and/or IL-17F mRNA

**Figure 7:**
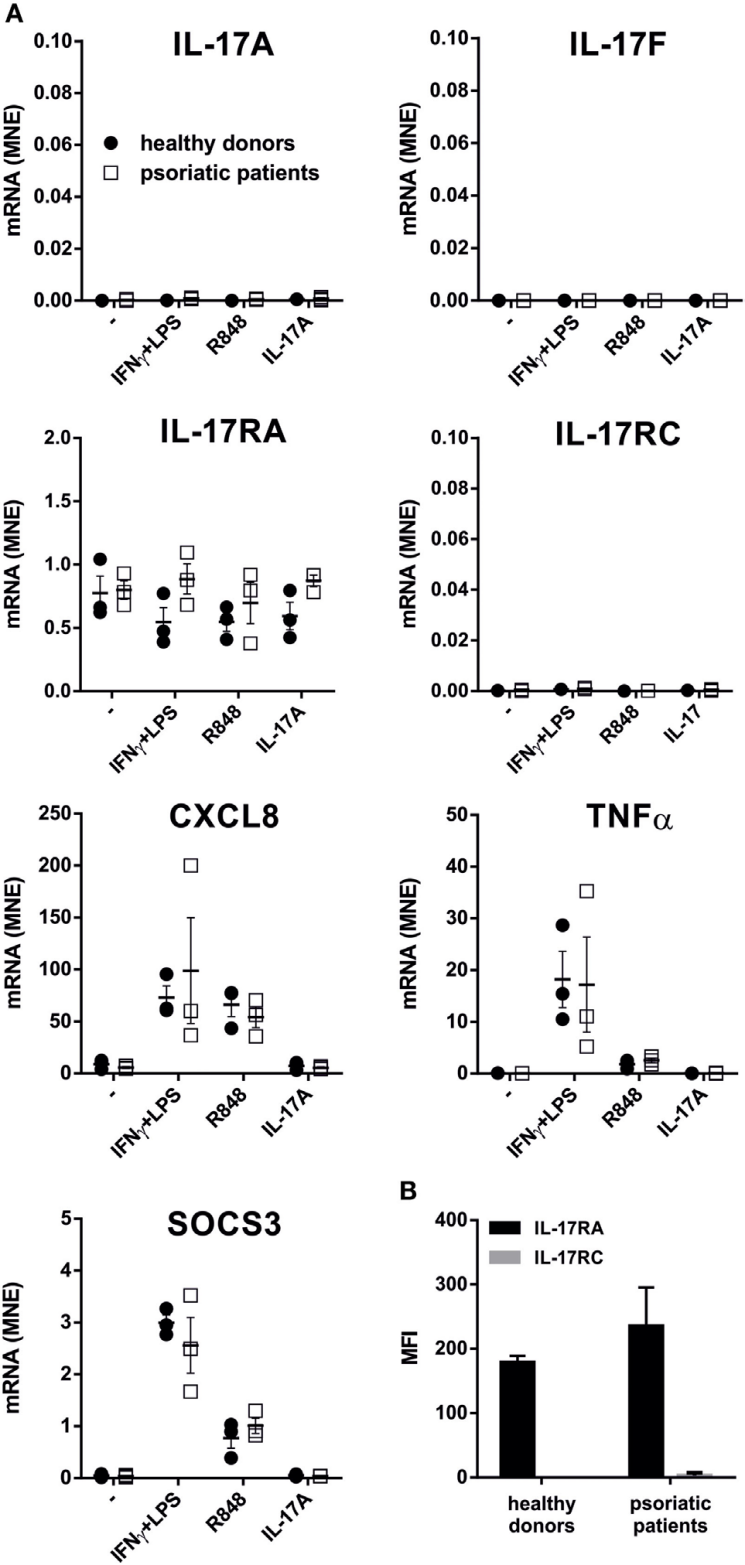
IL-17A, IL-17F, IL-17RA, IL-17RC, CXCL8, TNFα, and SOCS3 mRNA expression, as well as IL-17R surface expression, in neutrophils from patients with psoriasis. **(A)** Neutrophils isolated from healthy donors (HDs) (*n* = 3) or psoriatic patients (*n* = 3) were cultured for 20 h with 100 U/ml IFNγ plus 100 ng/ml LPS, 5 µM R848, or 500 ng/ml IL-17A to evaluate IL-17A, IL-17F, IL-17RA, IL-17RC, CXCL8, TNFα, and SOCS3 mRNA expression by reverse transcription quantitative real-time PCR. Gene expression data are depicted as mean normalized expression (MNE) units after GAPDH mRNA normalization. **(B)** Surface IL-17RA and IL-17RC expression evaluated by flow cytometry in human neutrophils from HDs or psoriatic patients. Values represent the mean ± SEM (*n* = 3). For the data of panels **(A,B)** no significant differences between HDs or psoriatic patients were observed by two-way ANOVA followed by Bonferroni’s post-test.

We subsequently investigated whether neutrophils isolated from patients with active psoriasis could express/produce IL-17A, IL-17F, and/or IL-17RC mRNA, either constitutively or upon incubation for 20 h with IFNγ plus LPS, R848, or IL-17A. As shown in Figure 7A, the latter was not the case, as psoriatic neutrophils behaved similarly to neutrophils from HDs. Psoriatic neutrophils did not also respond to IL-17A (Figure 7A), due to their lack of surface IL-17RC expression (Figure 7B). Nonetheless, psoriatic neutrophils fully responded to either R848 or IFNγ plus LPS, as they accumulated CXCL8, TNFα, and SOCS3 transcripts at levels comparable to those in HD neutrophils (Figure 7A).

### Commercial Anti-IL-17A Abs (AF-317-NA) Positively Stain Cytospins of Resting and Activated Neutrophils due to Their Non-Specific Recognition of Neutrophil Intracellular Proteins Different From IL-17A

**Figure 8:**
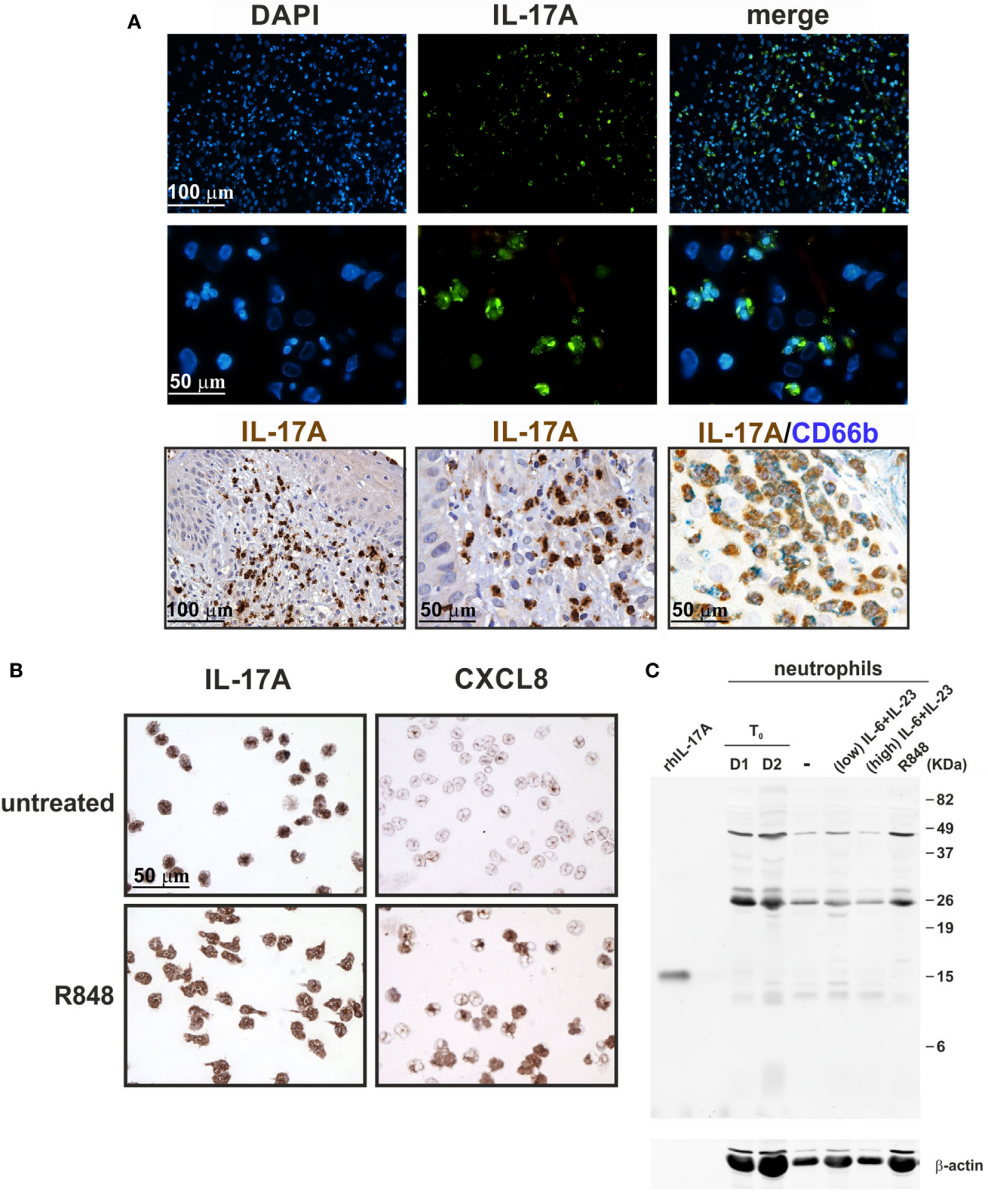
Staining human neutrophils by anti-IL-17A (AF-317-NA) polyclonal antibodies (Abs). **(A)** Immunofluorescence (top panels) and immunohistochemistry (lower panels) stainings of two FFPE cases of human pustular psoriasis using anti-IL-17A (AF-317-NA) and anti-CD66b Abs (as labeled). Top panels show DAPI, FITC channel, and merge to recognize neutrophil shape; lower panels show different magnification of IHC and double IHC to characterize IL-17A^+^ cells with the neutrophil marker CD66b. **(B)** Cytospins of neutrophils, either untreated (top panels) or treated with 5 µM R848 (bottom panels) for 3 h, were stained with anti-IL-17A (AF-317-NA, left panels) and anti-CXCL8 (right panels) Abs. Original magnification 200× [first row in **(A)** and left image in third row, scale bar 100 µm] and 400× [second row in **(A)**, center/right images in third row in **(A)**, as well as in **(B)**, scale bar 50 µm]. Images of the second row in **(A)** represent magnifications of images in first row. **(C)** AF-317-NA immunoblot of lysates from neutrophils either freshly isolated (T_0_, from two donors) or incubated for 3 h with or without 2 µg/ml IL-6 plus 0.2 µg/ml IL-23 (low), 20 µg/ml IL-6 plus 2 µg/ml IL-23 (high), or 5 µM R848. Recombinant human IL-17A (rhIL-17A) was used as positive control. Panels **(B,C)** display representative experiments out of two independent ones with similar results.

**Figure 9:**
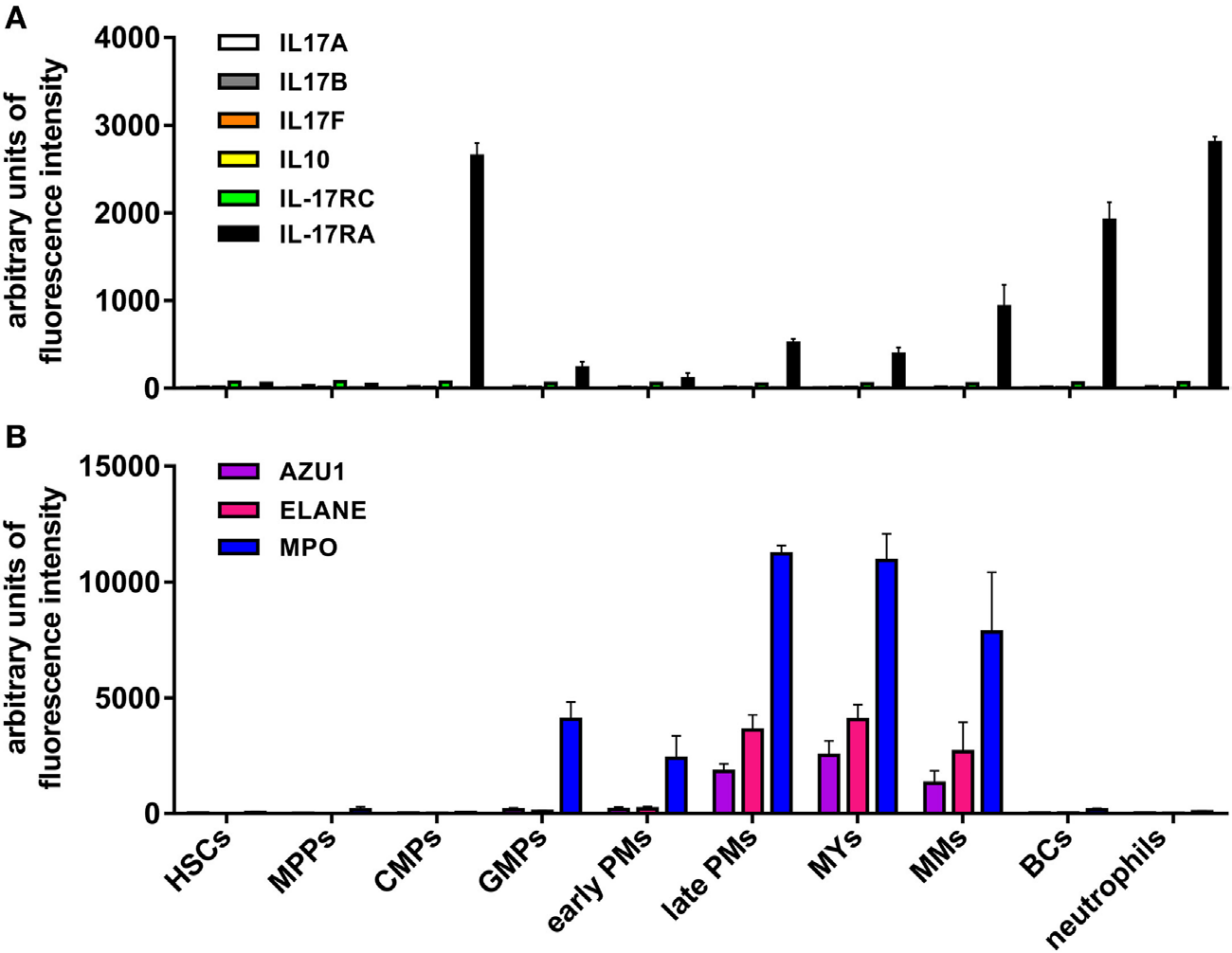
Levels of IL-17A, IL-17B, IL-17F, IL-10, IL-17RC, IL-17RA, azurocidin, neutrophil elastase, and myeloperoxidase (MPO) mRNA expression in neutrophils at different stages of maturation. mRNA expression data derive from Gene Expression Omnibus database (accession number GSE42519) (65). **(A)** IL-17A, IL-17B, IL-17F, IL-10, IL-17RC, and IL-17RA or **(B)** azurocidin (AZU1), neutrophil elastase (ELANE), and MPO mRNA expression levels were measured in the following cell types: hematopoietic stem cells (HSCs), multipotent progenitors (MPPs), common myeloid progenitors (CMPs), granulocyte-macrophage progenitors (GMPs), early and late promyelocytes (PMs), myelocytes (MYs), metamyelocytes (MMs), band cells (BCs), and bone marrow polymorphonuclear neutrophil granulocytes. Values represent the mean ± SEM as calculated from data of the biological replicates present in the database.

In additional experiments, cytospin slides of resting and R848-stimulated neutrophils were incubated with goat anti-human IL-17A AF-317-NA Abs, previously shown to stain neutrophils in pathological tissues (18–22, 25, 27, 28, 30–33, 35, 36, 41–43, 45, 47–50), as also confirmed by our IHC/IF staining of inflamed psoriatic tissue (Figure 8A). Consistently, neutrophil cytospin slides became strongly positive upon incubation with AF-317-NA, yet with no difference between resting or R848-activated neutrophils (Figure 8B). By contrast, immunostaining of the same cytospins slides with anti-human CXCL8 Abs showed a strong positivity only in R848-treated neutrophils (Figure 8B), thus excluding methodological artifacts. Not surprisingly, neutrophils from the same experiments were found totally negative for both IL-17A mRNA expression and IL-17A production once processed for RT-qPCR analysis and ELISA. The detection of IL-17A-positive neutrophils by IHC, in the absence of IL-17A mRNA, could be explained by the fact that the cytokine may be synthesized in bone marrow neutrophil precursors, at stages during which granule proteins, such as myeloperoxidase (MPO), elastase, and azurocidin 1, are formed (74). However, in transcriptomes made by Rapin et al., generated from cells isolated at different stages during granulopoiesis (65), we did not identify any IL-17A mRNA accumulation (Figure 9). In the same database, we not even detected IL-17RC and IL-10 mRNA (Figure 9), consistent with the inability of mature neutrophils to express them (12, 70). By contrast, we did find MPO, elastase, and azurocidin 1 mRNA expression only in transcriptomes of neutrophil precursors, as expected (74), thus validating the reliability of the database (65) (Figure 9). In any case, consistent with the absence of intracellular IL-17 (Figure 3A), immunoblots performed with AF-317-NA revealed that whole neutrophil lysates did not show any positive signal in correspondence of recombinant human IL-17A (rhIL-17A) molecular weight (MW) (Figure 8C). In these experiments, neutrophils were either freshly isolated (D1 and D2 in Figure 8C), or cultured for 3 h with or without R848, 2 µg/ml IL-6 plus 0.2 µg/ml IL-23 (low IL-6 plus IL-23 in Figure 8C), or 20 µg/ml IL-6 plus 2 µg/ml IL-23 (high IL-6 plus IL-23 in Figure 8C). By contrast, AF-317-NA strongly reacted in correspondence of neutrophil proteins displaying higher MW than that of rIL-17A, with no difference in signals among freshly isolated, stimulated, or untreated neutrophils (Figure 8C). While these data confirm the observations reported by Tamarozzi et al. (13), who also used mouse anti-IL-17A mAbs (#41802, from R&D) in addition to AF-317-NA, they are in contrast with Lin et al.’s findings (30) illustrating a constitutive IL-17A (but not IL-17F) expression in neutrophil lysates, as revealed by immunoblotting with #41802. Halwani et al. (23) too found constitutive IL-17A amounts in lysates of neutrophils from asthmatic patients, even increasing upon cell incubation with IL-21 and/or IL-23 for 18 h, as revealed by immunoblotting with unspecified Abs from R&D. However, since only portions of the immunoblots are shown in Halwani et al. (23) and Lin et al. (30) paper, it is not known whether additional proteins were recognized by Abs used. Whatever the case is, our experiments suggest that the positive staining of neutrophils detected by IHC and IF using AF-317-NA on cytospins and, possibly, tissue slides, stands for an IL-17A-unrelated binding(s) to neutrophils.

### Human Neutrophils Do Not Express/Produce IL-17B

**Figure 10:**
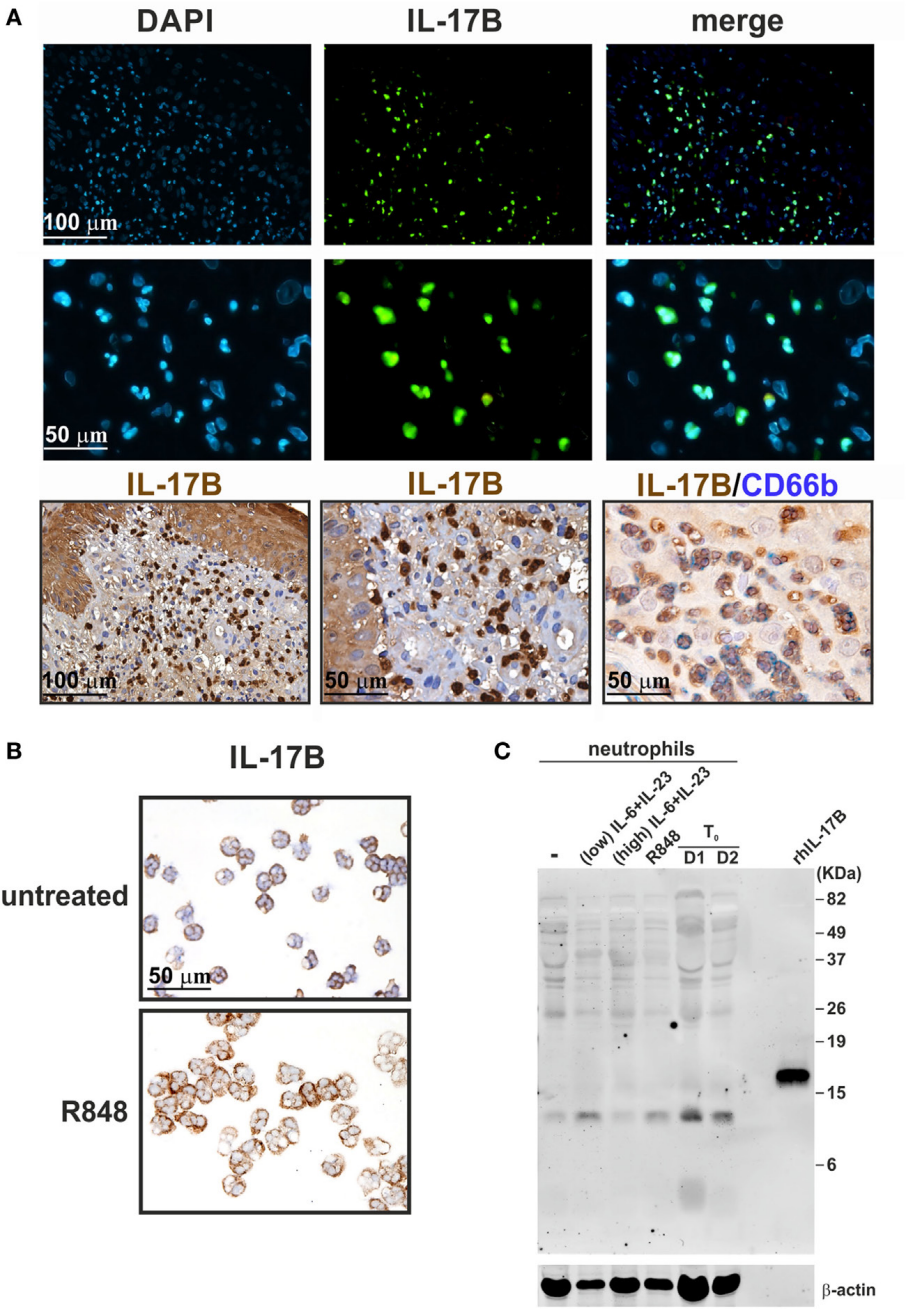
Staining human neutrophils by anti-IL-17B (AF1248) antibodies (Abs). **(A)** Immunofluorescence (top panels) and immunohistochemistry (lower panels) staining of two FFPE cases of human pustular psoriasis using anti-IL-17B (AF1248) and CD66b Abs (as labeled). Top panels show DAPI, FITC channel, and merge to recognize neutrophil shape; lower panels show different magnification of IHC and double IHC to characterize IL-17A^+^ cells with the neutrophil marker CD66b. **(B)** Cytospins of neutrophils incubated without (top panel) or with 5 µM R848 (bottom panel) for 3 h. Original magnification 200× [first row in **(A)** and left image in third row, scale bar 100 µm] and 400× [second row in **(A)**, center/right images in third row in **(A)**, as well as in **(B)**, scale bar 50 µm]. Images of the second row in **(A)** represent magnifications of images in first row. **(C)** AF1248 immunoblot of lysates from neutrophils either freshly isolated (T_0_, from two donors) or incubated for 3 h with or without 2 µg/ml IL-6 plus 0.2 µg/ml IL-23 (low), 20 µg/ml IL-6 plus 2 µg/ml IL-23 (high), or 5 µM R848. Recombinant human IL-17B (rhIL-17B) was used as positive control. Panels **(B,C)** display representative experiments out of two independent ones with similar results.

In a separate set of experiments, we also tested goat anti-IL-17B (AF1248) Abs that, in recent publications, have been shown to positively stain, by IHC and IF, neutrophils present in tissue samples from RA (75) and colon carcinoma (CCR) (76) patients. Consistently, we found that also neutrophils present in inflamed psoriatic tissue were strongly detectable by IHC and IF stainings with AF1248 (Figure 10A). On cytospin slides, AF1248 stained neutrophils isolated from the blood of HDs and incubated for 3 h with or without 5 µM R848 in an equivalent manner (Figure 10B). However, by immunoblotting of whole lysates prepared from neutrophils treated with R848 or IL-6 plus IL-23, AF1248 did not recognize any protein corresponding to the rhIL-17B MW (Figure 10C). These negative observations were also confirmed by measurement of intracellular, as well as, released IL-17B by two commercial ELISA (see Materials and Methods). Accordingly, no antigenic IL-17B could be measured in supernatants and whole lysates from neutrophils incubated with 5 µM R848 with or without 1,000 U/ml IFNα, 100 µg/ml LPS with or without 100 U/ml IFNγ, 2/20 μg/ml IL-6 plus 2 µg/ml IL-23 (data not shown), in agreement with the lack of IL-17B mRNA induction. Detectable IL-17B levels were, however, measured in lysates of human cerebral cortex (68), demonstrating that our two IL-17B ELISA kits were sensitive enough. Altogether, our data indicate that, similarly to the case of AF-317-NA, the positive stainings of neutrophils in cytospin slides and, possibly, tissue samples by AF1248, likely stand for an IL-17B-unrelated, non-specific, recognition occurring in human neutrophils.

## Discussion

In this study, we have reinvestigated in-depth whether human neutrophils produce IL-17A, IL-17B, IL-17F, and IL-17A/F *in vitro*. According to the literature, in fact, information on such an issue appears discordant, as the majority of papers sustain that human neutrophils do express/produce IL-17A (18–53), while a minority fail to detect it (12–17). This issue is even more critical if one takes into account that also the capacity of murine neutrophils to produce IL-17A, shown in a variety of mouse models of infectious and autoimmune inflammation (24, 39, 40, 77–81), has been recently questioned (82, 83). Preclinical models evidencing neutrophil-derived IL-17 as pathogenic in diseases might be, in fact, prematurely taken as proof-of-concept for immediate translational applications in humans.

Herein, by using multiple methodological approaches (RT-qPCR, ChIP, ChIP-seq, ELISA, intracellular staining, and immunoblotting), we confirm and greatly extend our previous findings (12) showing that highly purified (>99.7%) populations of human neutrophils, either resting or activated by a variety of stimulatory conditions, including TLR and dectin ligands, fungal PAMPs and cytokines, used singly or in combinations, neither express IL-17A, IL-17F, IL-17B, IL-17C, IL-17D, and IL-17E mRNA nor produce IL-17A, IL-17F, IL-17A/F, and IL-17B *in vitro*. Similarly, we show that also neutrophils isolated from patients with active psoriasis do not express IL-17F, IL-17B, IL-17C, IL-17D, and IL-17E as well as IL-17RC mRNA when activated by R848, IFNγ plus LPS, and IL-17A *in vitro*. In such regard, RNA-Seq experiments made by Tamarozzi et al. (13), using neutrophils isolated by negative-selection (>99.9% pure) from HDs or RA patients (as we do), then treated for 1 h with a range of inflammatory cytokines (TNFα, GM-CSF, G-CSF, IL-6, IL-1β, CXCL8, IFNα, and IFNγ), also failed to detect any of the mRNA for IL-17 cytokine family. By contrast, Yamanaka et al. (15) have been recently reported the presence of constitutive IL-17A transcripts in neutrophils from HDs and psoriasis patients isolated by density gradient cell separation (92% purity). However, when the same cell populations were further purified by magnetic sorting (reaching a 99% purity), they were found totally devoid of IL-17A mRNA (15), indicating that contaminating monocytes/lymphocytes were actually responsible for the IL-17A mRNA expression in unsorted “neutrophils.” Needless to say that Yamanaka et al.’s observations (15) are example of a notion that we have been always recommending in our studies (56, 59, 84), namely the requirement of using highly purified cell populations if one wants to obtain correct results when examining neutrophil gene expression or neutrophil-derived cytokines.

Interestingly, other studies confirm that human neutrophils do not constitutively contain IL-17A transcripts (13, 24, 29, 30, 35, 39, 40, 44), including those ultimately showing a concurrent positivity for IL-17A protein, as revealed by intracellular flow cytometry (24, 39, 40), ELISA (24, 39), confocal microscopy (39), or IHC (29). Some authors (30, 35) speculated that the absence of IL-17A mRNA in mature neutrophils indicates that the cytokine is synthesized in bone marrow neutrophil precursors, at the stages when granule proteins are formed (74). However, we would exclude such a hypothesis, as our analysis of transcriptomes generated from all types of bone marrow cell populations (65) failed to identify an IL-17A mRNA accumulation at any stage of neutrophil maturation.

We were unable to detect IL-17A and IL-17F mRNA/production/release even by human neutrophils incubated with IL-6 plus IL-23, in contrast to what repeatedly found (23, 24, 29, 39, 40). In our experiments, neutrophils did, however, respond to IL-6 plus IL-23 in terms of STAT3 phosphorylation and SOCS3 mRNA induction, indicating that the two cytokines are effectively stimulatory for neutrophils. It is intriguing that, apart from Halwani et al. (23), who found that either 20 ng/ml IL-6 or 20 ng/ml IL-23, singly used, directly induced IL-17A mRNA and protein in a fraction of neutrophils from asthmatic patients, other groups highlighted the necessity to use at least 20 µg/ml IL-6 plus 2 µg/ml IL-23 (29, 39, 40) (as we did). In this context, the paper by Hu et al. (24), based on the use of neutralizing Abs and pharmacological inhibitors, identified endogenous IL-6 and IL-23 as indirect inducers of IL-17A expression in a fraction of neutrophils either infected with *Mycobacterium tuberculosis* (MTB), or stimulated with LPS or Pam3CSK4. In this latter study, however, IL-6 and IL-23 levels corresponded to 1 ng/ml at the best. Herein, we failed to detect IL-17A mRNA expression and production in neutrophils incubated with either LPS or Pam3CSK4, even if it is true that they produce IL-6 (62) and IL-23 (our unpublished observations). Whether stimulation of neutrophils with MTB effectively promotes IL-17A expression *via* endogenous IL-6 and IL-23 remains to be verified. However, no IL-17A, IL-17B, IL-17C, or IFNγ secretion from *Mycobacterium bovis* Bacille-Calmette Guerin (BCG)-stimulated neutrophils was recently reported (14). It should be also remarked that the purity of neutrophils in studies showing an IL-17 production in response to IL-6 plus IL-23 (23, 29, 39, 40), reported to be >96% at the best (29), does not sufficiently secure fully genuine results at least in our opinion.

Nevertheless, we investigated potential mechanisms helping to clarify whether human neutrophils respond to IL-6 plus IL-23 in terms of IL-17A expression or not. ChIP assays revealed that, in resting, as well as in IL-6 plus IL-23-stimulated, neutrophils, but not in Th17 cell lines, the *IL17A* locus does not contain any H3K4me1 and H3K27Ac, which are two histone marks that are usually present in those genomic regions that act as active enhancers (85). On the other hand, the levels of H3K27Ac were found increased at the SOCS3 promoter of neutrophils incubated with IL-6 plus IL-23, consistent with the potentially inducible SOCS3 mRNA transcription. Notably, the complete absence of H3K4me1 at the *IL17A* locus of neutrophils is particularly informative, since such a histone modification is known to precede very early, but time-consuming (86), events necessary for the assembly of the transcriptional machinery, including nucleosomal depletion, H3K27Ac deposition, and enhancer activation (85). Based on our data, it appears that the chromatin at the *IL17A* locus of human neutrophils likely displays a closed conformation, inaccessible to transcription factors and, consequently, RNA polymerase, ultimately preventing IL-17A mRNA transcription in resting as well as stimulated neutrophils. It is thus very unlikely that H3K4me1 modification could be induced within 1 h, e.g., the time-point at which IL-17 mRNA expression in IL-6 plus IL23-stimulated neutrophils has been observed (29, 39, 40). Obviously, this does not exclude that there could exist some stimulatory conditions able to modify the chromatin at the *IL17A* or *IL17F* loci of human neutrophils.

A variety of studies report the presence of IL-17A^+^-neutrophils in sample tissues from many diseases, including psoriasis (20, 25, 30, 32, 35, 49), skin inflammation (27), bullous pemphigoid (28), hidradenitis suppurativa (50), fungal keratitis (26), RA (31, 75), ankylosing spondylitis (18), systemic lupus erythematosus (41, 52), human ANCA-associated glomerulonephritis (47), cystic fibrosis (19, 36, 44), nasal polyps (53), chronic obstructive pulmonary disease (22), lung tissues during bacterial pneumonia (46), alcoholic liver diseases (48), acute renal allograft rejection (42), atherosclerotic plaques (21), cutaneous T cell lymphoma lesions (45), gastric cancer (29), cervical cancer (33), and prostate cancer (51), as revealed by IHC, IF, or intracellular flow cytometry using various commercial anti-IL-17A Abs. Not surprisingly, results occasionally appear discordant. For example, while Moran et al. (31) reported IL-17A-positive synovial tissue neutrophils using the AF-317-NA, van Baarsen et al. (16) show that synovial tissue neutrophils from arthritis patients are not stained by another antibody, namely #41802. By IHC experiments using AF-317-NA, we too detected IL-17A^+^-neutrophils not only in skin sections of psoriasis patients but also in cytospin slides of neutrophils isolated from HDs and incubated for 3 h with or without R848, at similar levels. By contrast, we found that whole lysates of the same neutrophil populations displayed major signals at levels of proteins having MW not corresponding to that of IL-17A when immunoblotted with AF-317-NA. Our findings substantially confirm the observations previously made by Tamarozzi et al. (13) who also did not detect any IL-17A expression in highly pure populations of neutrophils (99.9%) by using a variety of assays including RT-qPCR, RNA-seq, western blot and ELISA, despite of finding IL-17A^+^-neutrophils in *Wolbachia Onchocerca volvulus*-positive nodules by IHC using AF-317-NA. Notably, by immunoprecipitation experiments followed by mass spectrometry, Tamarozzi et al. (13) also uncovered that both AF-317-NA and #41802 bind to several proteins expressed in granules (including MPO, lactoferroxin, and lysozyme C) and cytoskeleton (such as keratin and profilin) of neutrophils, while other anti-human IL-17A Abs (sc-6077 from Santa Cruz, and PRS4877 from Sigma) were found to recognize multiple non-specific bands in neutrophil immunoblots (13). All in all, data suggest that the IL-17A-positivity of human neutrophils detected by AF-317-NA and #41802 is, at least *in vitro*, likely an artifact. Whether these or other anti-IL-17A Abs, including sc-7927 (from Santa Cruz) (33, 43), ab9565 (from Abcam) (37), ab136668 (from Abcam) (46), 500-P07 and 500-P07G (from Peprotech) (43), and eBio64Dec17 (from eBioscience) (20, 26, 43), are instead reliable in specifically detecting IL-17A^+^-neutrophils in tissue samples should be more convincingly established. For instance, in models of skin inflammation resembling psoriasis (27), accumulated neutrophils stained by AF-317-NA were shown to express IL-17 mRNA transcripts. In other studies, tissue neutrophil staining by AF-317-NA was blocked after antibody pre-adsorption with rIL-17A (18, 47), or confirmed by costaining of the same section by eBio64DEC17 (47). It is worth recalling that neutrophils express high levels of IL-17RA (12) that could in theory bind exogenously derived IL-17A, consequently leading to a positive signal in IHC or IF experiments without actual intracellular IL-17 production (87), as observed in the case of mast cells (88). Whatever the case is, we would recommend to always validate by multiple investigation methods an eventual detection of IL-17A-positive neutrophils exclusively by IHC, or IF or intracellular flow cytometry (18, 19, 21, 22, 28, 32–34, 36–38, 42, 43, 48–52).

Similar concerns can be made for the, to date, reported IL-17B expression by human neutrophils. Accordingly, IL-17B has been detected in neutrophils infiltrating the synovial membrane of RA patients (75) and the stroma of CCR cancer (76) by IHC/IF, as well as in freshly isolated neutrophils by immunoblotting (75), in all cases using #AF1248 Abs. We also detected IL-17B-positive neutrophils in psoriasis plaques and cytospin slides of freshly isolated neutrophils by IHC using #AF1248. However, we could not measure any IL-17B in lysates of freshly isolated/activated neutrophils either by using two different commercial ELISA or by #AF1248 immunoblotting. In the latter experiments, many proteins with MW different from that of rIL-17B were recognized by #AF1248, thus invalidating at least the cytospin results. Intriguingly, Kouri et al. (75) did detect IL-17B protein in lysates of neutrophils (95% pure), by both ELISA and immunoblotting using #AF1248. However, these authors showed only a portion of the western blot (75), thus rendering impossible to know whether additional major proteins were recognized by #AF1248. Curiously, we, Tamarozzi et al. (13) and Kouri et al. (75), all found that human neutrophils do not transcribe IL-17B mRNA under resting or activating condition. Furthermore, no IL-17B secretion from BCG-stimulated neutrophils was recently shown (14). In such regard, Koury et al. (75) suggested that IL-17B is synthesized only at the promyelocyte and myelocyte stage in the bone marrow, disappearing in mature neutrophils. However, our analysis of transcriptomes generated from all types of bone marrow cell populations (65) revealed that, similarly to IL-17A, also IL-17B is never transcribed during the different stages of neutrophil maturation. Altogether, data suggest that human neutrophils do not express IL-17B *in vitro*. They also suggest that the positive staining of neutrophils by IHC using AF1248 is likely due to a non-specific, IL-17B independent, binding of these Abs.

In conclusion, data shown in this study are consistent with the notion that human neutrophils are unable to express and produce IL-17A, IL-17B, or IL-17F *in vitro*.

## Ethics Statement

This study was carried out in accordance with the recommendations of Ethic Committee of the Azienda Ospedaliera Universitaria Integrata di Verona (Italy). All the experimental protocols were approved by the Ethic Committee and all subjects gave written informed consent in accordance with the Declaration of Helsinki.

## Author Contributions

All authors were involved in discussing and drafting the article, approved the final version to be published, and had full access to all data, taking responsibility for their integrity and analysis accuracy. In particular, NT, FA-S, SG, EG, SL, LG, and FC performed the experiments; FA-S, FB-A, NT, SL, WV, and MC analyzed the results; GG provided patients; and FS, NT, AM, WV, and MC conceived the experiments and wrote the paper.

## Conflict of Interest Statement

The authors declare that the research was conducted in the absence of any commercial or financial relationships that could be construed as a potential conflict of interest.

## Footnotes

^1^http://www.gene-quantification.de/download.html (Accessed: February 10, 2018).

^2^http://egg2.wustl.edu/roadmap/web_portal/processed_data.html (Accessed: February 10, 2018)
