# Supplementary material for: A Reappraisal on the Potential Ability of Human Neutrophils to Express and Produce IL-17 Family Members In Vitro: Failure to Reproducibly Detect It
Source: Front Immunol. 2018 Apr 17;9:795. doi: 10.3389/fimmu.2018.00795 (PMC5913333; doi:10.3389/fimmu.2018.00795)
Supplement: Supplementary file 1 [file Presentation_1.PDF]

Supplementary Material

A reappraisal on the ability of human neutrophils to express and produce IL-17 family members

Nicola Tamassia[1], Fabio Arruda-Silva[1], Federica Calzetti[1], Silvia Lonardi[2], Sara Gasperini[1], Elisa Gardiman[1], Francisco Bianchetto-Aguilera[1], Giampiero Girolomoni[3], Alberto Mantovani[4], William Vermi[2], Marco A. Cassatella[1*]

*** Correspondence:** Prof. Marco A. Cassatella, Section of General Pathology, Department of Medicine, University of Verona, Verona, Italy, marco.cassatella@univr.it

Supplementary table 1 | List of human primer sets utilized for RT-qPCR experiments

**Gene**	**Sequence**	
	**forward primers**	**reverse primers**
GAPDH	AACAGCCTCAAGATCATCAGC	GGATGATGTTCTGGAGAGCC
RPL32	AGGGTTCGTAGAAGATTCAAGG	GGAAACATTGTGAGCGATCTC
SOCS3	GGCCACTCTTCAGCATCTC	ATCGTACTGGTCCAGGAACTC
IL-1ra	TTCCTGTTCCATTCAGAGACGAT	AATTGACATTTGGTCCTTGCAA
IL-17A	CTCATTGGTGTCACTGCTACTG	CCTGGATTTCGTGGGATTGTG
IL-17B	ACAACCTGCTGTTTCTTCTTACC	ACCATCTCCTCGATGTTCCTC
IL-17C	GCTACTCGGCTGAGGAACTG	GTGTCCACACGGTATCTCCA
IL-17D	CTACTGGAGCAGCTGTACG	GTCGTAGGAGATTCTGTAGGC
IL-17E	TGGAGATATGAGTTGGACAGAG	GCTAAGGAAACACGGTACAG
IL-17F	CTGGAATTACACTGTCACTTGG	GAGATGTCTTCCTTTCCTTGAG
IL-17RA	AGACACTCCAGAACCAATTCC	TCTTAGAGTTGCTCTCCACCA
IL-17RC	GTCACTGTGGACAAGGTTCTC	CTCCAACAGTAGCACATCGTC
TNFα	GAGCACTGAAAGCATGATCC	CGAGAAGATGATCTGACTGCC
CXCL8	CTGGCCGTGGCTCTCTTG	CCTTGGCAAAACTGCACCTT

Supplementary table 2 | List of human primer sets utilized for qPCR of ChIP assays

ChIP primer name	Location (relative to the gene TSS)	Sequence	
		forward primers	reverse primers
IL-17A#1	-30260 to -30119	CACAAAACCGCAGGTACTCAG	TCACCACAAAGCCCACAAAG
IL-17A#2	-177 to -53	TGCCCTTCCCATTTTCCTTC	TCCTTCTGTGGTCACTTACG
IL-17A#3	+704 to +898	TAGCACCAACAGCACTTCTAGC	CAGCACATGCATCATTGTCAG
IL-17F#1	-19472 to -19371	AAGACATGACCCCCAGAGATC	GTTTTCCTTGAGAGCAATCGTG
IL-17F#2	-223 to -132	CAATGGGGGTGGAAGTAGG	CCGAAGGGGAACAAAAGGG
SOCS3	-113 to -45	TCTCTGCTGCGAGTAGTGAC	CCGCCCCCCGATTCCTGGA
PRL	+386 to +506	AGGGAAACGAATGCCTGATT	GCAGGAAACACACTTCACCA

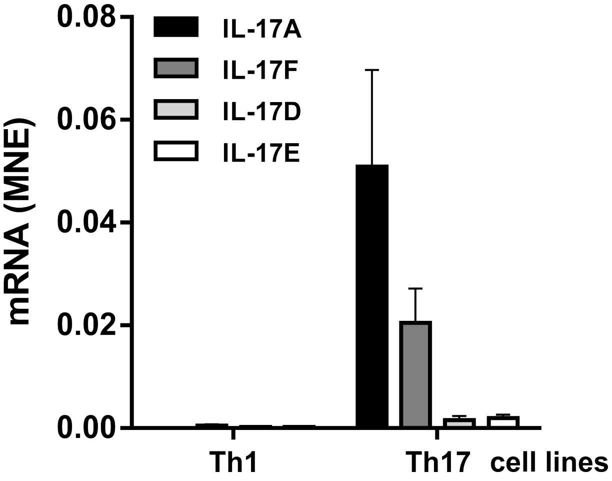

Supplementary figure 1 | Expression of IL-17A, IL-17F, IL-17D and IL-17E in human Th1 and Th17 cell lines.

mRNA of Th1 and Th17 cell lines was extracted to evaluate IL-17A, IL-17F, IL-17D and IL-17E mRNA expression by RT-qPCR. Gene expression data are depicted as mean normalized expression (MNE) units after GAPDH mRNA normalization (means ± SEM).

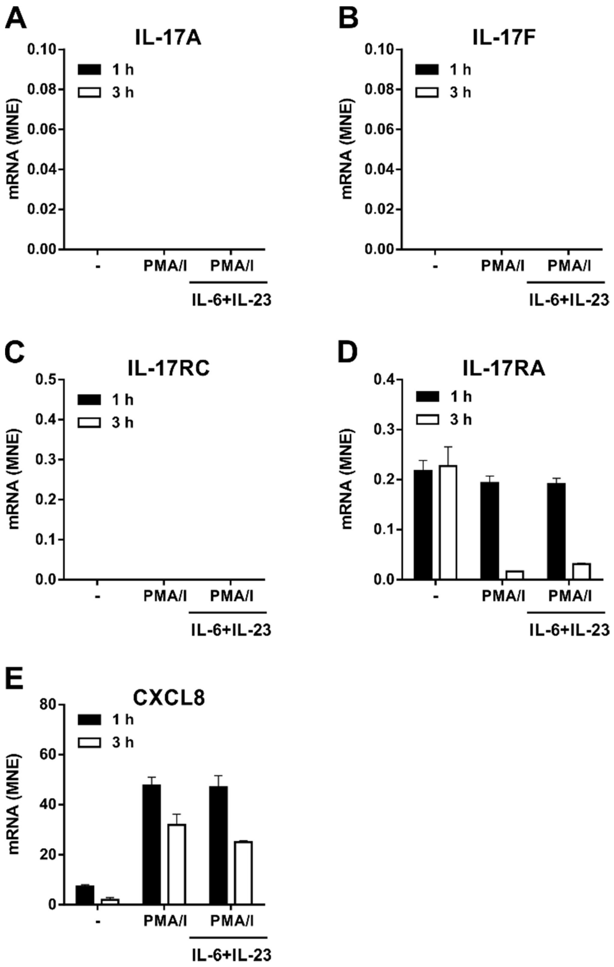

Supplementary figure 2 | No induction of IL-17A, IL-17F and IL-17RC expression in human neutrophils incubated with PMA/ionomycin

Neutrophils (5 × 10^6/ml) were incubated with or without 20 μg/ml IL-6 plus 2 μg/ml IL-23 and then cultured for additional 1 or 3 hours in the presence or not of 20 μg/ml PMA plus 1 μg/ml ionomycin. Neutrophils were then harvested for mRNA extraction to evaluate IL-17A **(A)**, IL-17F **(B)**, IL-17RC **(C)**, IL-17RA **(D)** and CXCL8 **(E)** mRNA expression by RT-qPCR. Gene expression data are depicted as mean normalized expression (MNE) units after GAPDH mRNA normalization (means ± SEM).

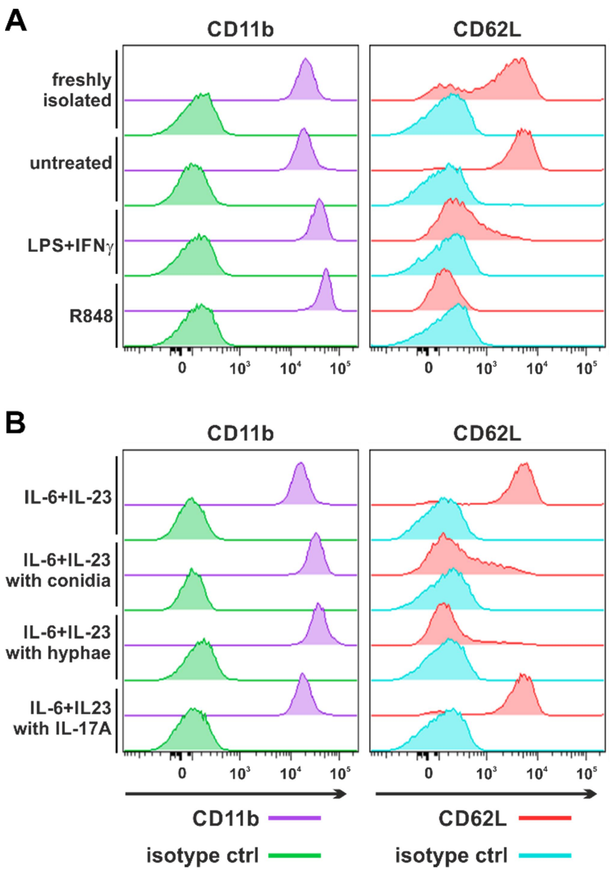

Supplementary figure 3 | Expression of surface CD11b and CD62L in neutrophils activated under various experimental conditions.

Expression of surface CD11b (left panel) and CD62L (right panel) was evaluated by flow cytometry in neutrophils either freshly isolated or cultured for 3 h without or with 100 U/ml IFNγ plus 100 ng/ml LPS, 5 µM R848 **(A)**, 20 µg/ml IL-6 plus 2 µg/ml IL-23 alone or in the presence of inactivated *A. fumigatus* conidia, hyphae or 500 ng/ml rIL-17A (**B**). Graphs depict a representative experiment out of three independent ones with similar results. Histograms show staining by specific and isotype control Abs, respectively, for each stimulatory condition.

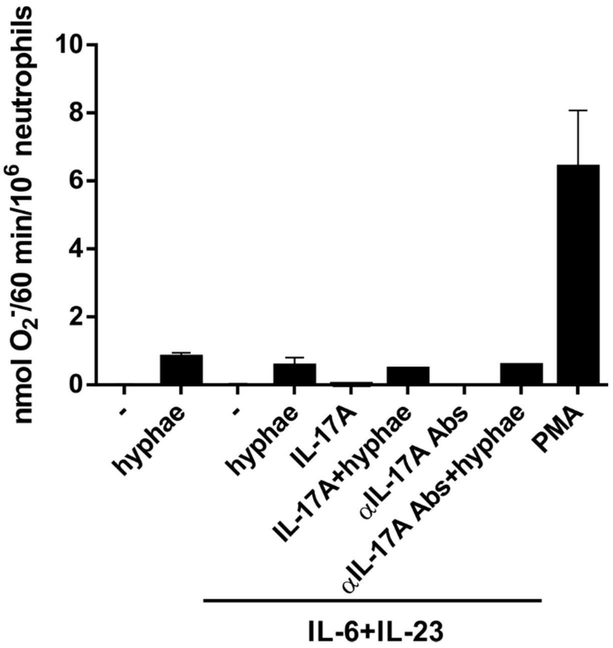

Supplementary figure 4 | Superoxide anion production by human neutrophils stimulated by inactivated hyphae from *A. fumigatus* in combination with IL-6 plus IL-23, IL-17A or secukinumab.

Neutrophils (5×10^6/ml) were preincubated with or without 20 µg/ml IL-6 plus 2 µg/ml IL-23 for 1 h and then treated for 1 more hour with inactivated hyphae from *A. fumigatus*, in combination or not with 500 ng/ml IL-17A or 10 µg/ml anti-IL-17A neutralizing mAb (secukinumab). As control, neutrophils were also stimulated with 20 ng/ml PMA for 1 h. Graph depicts a representative experiment out of three independent ones with similar results.
